# Study on the Alkylation Reactions of *N*(7)-Unsubstituted 1,3-Diazaoxindoles

**DOI:** 10.3390/molecules22050846

**Published:** 2017-05-19

**Authors:** Eszter Kókai, Judit Halász, András Dancsó, József Nagy, Gyula Simig, Balázs Volk

**Affiliations:** 1 Directorate of Drug Substance Development, Egis Pharmaceuticals Plc., P.O. Box 100, 1475 Budapest, Hungary; ekokai@mail.bme.hu (E.K.); halasz.judit@egis.hu (J.H.); dancso.andras@egis.hu (A.D.); simig.gyula@egis.hu (G.S.); 2 Department of Organic Chemistry and Technology, Budapest University of Technology and Economics, P.O. Box 91, 1521 Budapest, Hungary; jozsefnagy@mail.bme.hu; 3 Department of Materials Technology, GAMF Faculty of Engineering and Computer Science, Pallasz Athéné University, P.O. Box 700, 6001 Kecskemét, Hungary

**Keywords:** alkylation, lithiation, regioselectivity, 1,3-diazaoxindoles, by-products

## Abstract

The chemistry of the 5,7-dihydro-6*H*-pyrrolo[2,3-*d*]pyrimidin-6-one (1,3-diazaoxindole) compound family, possessing a drug-like scaffold, is unexplored. In this study, the alkylation reactions of *N*(7)-unsubstituted 5-isopropyl-1,3-diazaoxindoles bearing various substituents at the *C*(2) position have been investigated. The starting compounds were synthesized from the *C*(5)-unsubstituted parent compounds by condensation with acetone and subsequent catalytic reduction of the 5-isopropylidene moiety. Alkylation of the thus obtained 5-isopropyl derivatives with methyl iodide or benzyl bromide in the presence of a large excess of sodium hydroxide led to 5,7-disubstituted derivatives. Use of butyllithium as the base rendered alkylation in the *C*(5) position possible with reasonable selectivity, without affecting the *N*(7) atom. During the study on the alkylation reactions, some interesting by-products were also isolated and characterized.

## 1. Introduction

**Figure 1:**
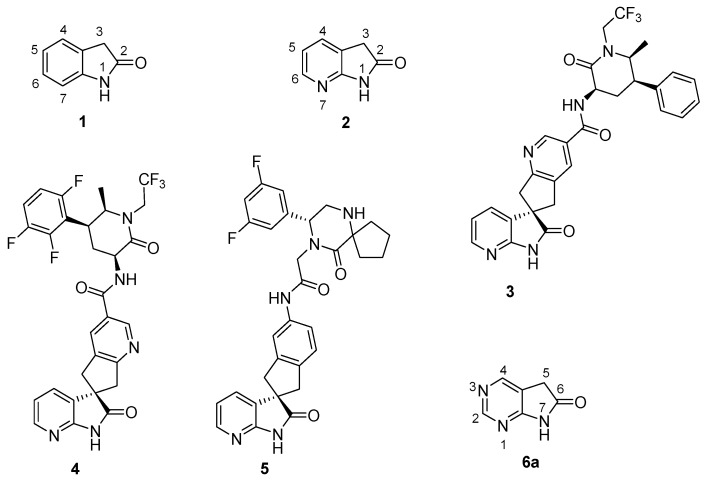
Oxindole **1**, 7-azaoxindole **2**, and drug candidates with a 7-azaoxindole skeletons: ubrogepant (**3**, MK-1602), MK-8031 **4**, MK-3207 **5**; and 1,3-diazaoxindole **6a**.

The biological activity of 1,3-dihydro-2*H*-indol-2-one (oxindole, **1**, Figure 1) derivatives is best demonstrated by launched drugs possessing this skeleton: the dopamine agonist ropinirole [1,2] for the treatment of Parkinson's disease and restless leg syndrome; the atypical antipsychotic ziprasidone [3]; and two oncology drugs from the tyrosine kinase inhibitor family, sunitinib [4] and the recently launched nintedanib [5]. The 7-aza analogue of oxindole, 1,3-dihydro-2*H*-pyrrolo[2,3-*b*]pyridin-2-one (7-azaoxindole, **2**) is also a skeleton of biological importance. Oral calcitonin gene-related peptide (CGRP) antagonists ubrogepant (MK-1602, **3**) [6] and MK-8031 (**4**) [6] are in phase III clinical development, while MK-3207 (**5**) [7,8,9] had been in phase II, all three for the acute treatment of migraine attacks (Figure 1).

Bioisosteric replacement of the aromatic carbon atom *C*(5) of 7-azaoxindole (**2**) with a nitrogen atom, i.e., that of the pyridine ring by a pyrimidine nucleus [10,11,12] formally leads to 5,7-dihydro-6*H*-pyrrolo[2,3-*d*]pyrimidin-6-one (**6a**, 1,3-diazaoxindole, Figure 1).

**Scheme 1:**
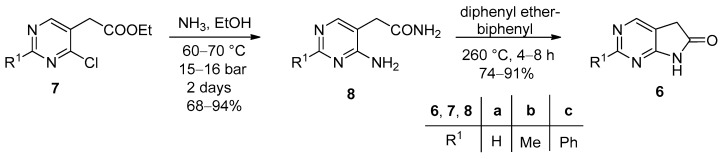
Synthesis of 5,7-dihydro-6*H*-pyrrolo[2,3-*d*]pyrimidin-6-ones (**6**, 1,3-diazaoxindoles).

Recently we have reported a new synthetic route to 1,3-diazaoxindoles **6** (Scheme 1). Treatment of ethyl 2-(4-chloropyrimidin-5-yl)acetate derivatives **7** with ethanolic ammonia at 60–70 °C afforded the corresponding 2-(4-aminopyrimidin-5-yl)acetamides **8**, which were finally cyclized to the target compounds **6** in a mixture of diphenyl ether and biphenyl at 260 °C [13].

**Scheme 2:**
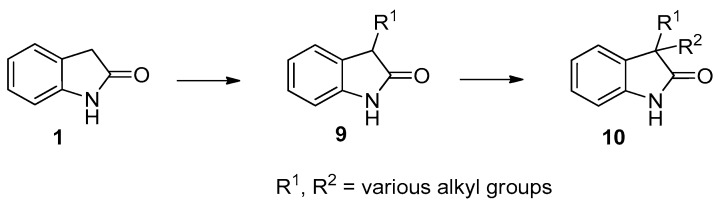
Alkylation of oxindole (**1**) to 3-monoalkyloxindoles **9** and subsequent alkylation to give 3,3-dialkyloxindoles **10**.

In continuation of our studies on the monoalkylation at the *C*(3) position of *N*-unprotected oxindole (**1**) [14,15,16] to give 3-alkyloxindoles **9** and on the selective introduction of the second alkyl group, without *N*-alkylation to afford *N*-unprotected 3,3-dialkyloxindoles **10** (Scheme 2) [17], we now aimed to elaborate a method for the introduction of two different alkyl groups into the structurally related carbonyl-adjacent *C*(5) site of *N*-unprotected 1,3-diazaoxindoles **6**.

**Scheme 3:**
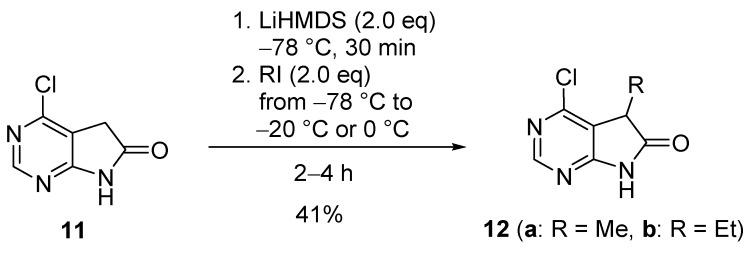
*C*(5)-Monoalkylation of 4-chloro-1,3-diazaoxindole (**11**).

A comprehensive literature search revealed that no systematic study had been carried out on the *C*(5)-monoalkylation or *C*(5)-dialkylation of 1,3-diazaoxindoles **6**. Alkylation of the parent compound **6a** (Scheme 1) itself is unprecedented, and only some special examples are described in patent applications as discussed below. *C*(5)-Monoalkylation of 4-chloro-1,3-diazaoxindole (**11**, Scheme 3), unsubstituted at the *C*(5) and *N*(7) atoms, was performed by Shepherd et al. using lithium bis(trimethylsilyl)amide (LiHMDS, 2.0 eq.) as the base in tetrahydrofuran (THF), with methyl or ethyl iodide (MeI or EtI, 2.0 eq.) to give the 5-methyl and 5-ethyl derivatives **12a** and **12b**, respectively, in both cases with 41% yield [18].

**Scheme 4:**
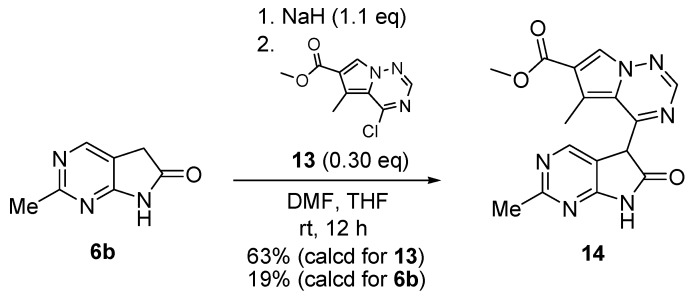
*C*(5)-Monoalkylation of 2-methyl-1,3-diazaoxindole (**6b**).

*C*(5)-Monosubstitution of 2-methyl-1,3-diazaoxindole (**6b**, Scheme 4) was carried out with chloro-substituted pyrrolotriazine **13** (0.30 eq.) in the presence of sodium hydride (1.1 eq.) to afford pyrrolopyrimidinyl-pyrrolotriazine **14** in 19% yield (calculated for **6b**) [19].

**Scheme 5:**
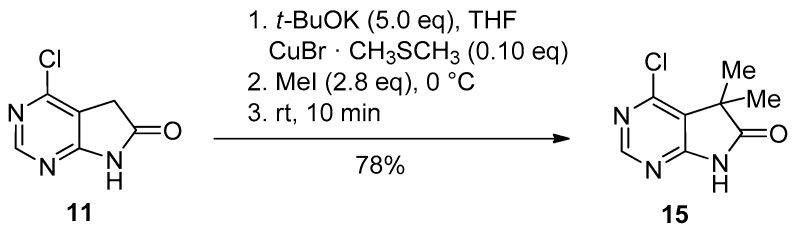
*C*(5)-Dimethylation of 4-chloro-1,3-diazaoxindole (**11**).

*C*(5)-Dialkylation of 4-chloro derivative **11** with potassium *tert*-butoxide (*t*-BuOK, 5.0 eq.) and MeI (2.8 eq.) in the presence of dimethyl sulfide and copper(I)bromide between 0 °C and ambient temperature gave 5,5-dimethyl derivative **15** in 78% yield (Scheme 5) [19].

**Scheme 6:**
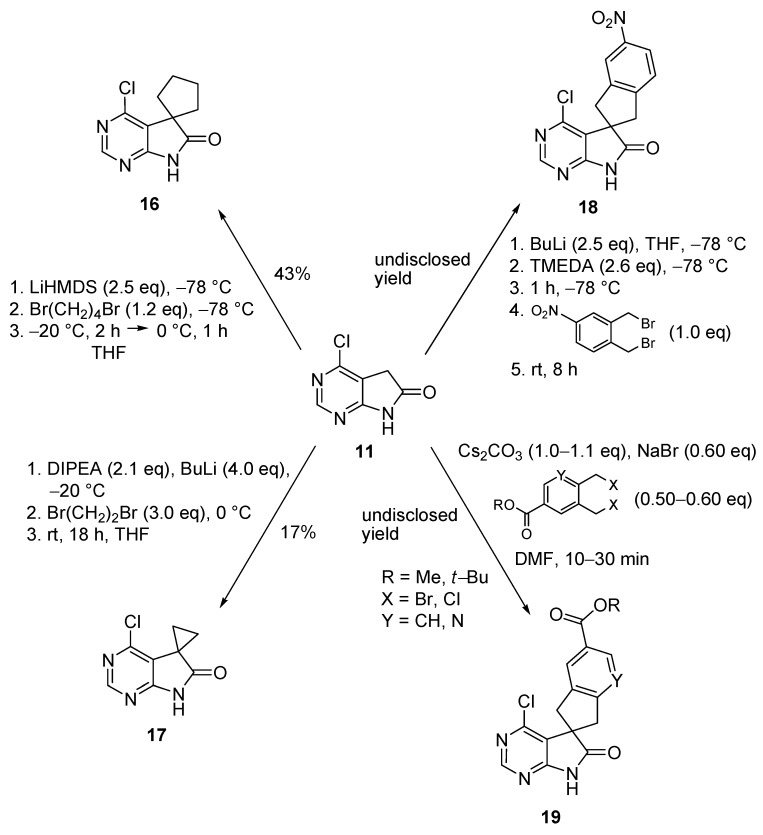
*C*(5)-Alkylation of 4-chloro-1,3-diazaoxindole (**11**) leading to spiro products **16**–**19**.

When the alkylation of **11** was performed with bis(haloalkyl) compounds, the corresponding spiro products **16**–**19** were obtained in low or undisclosed yields (Scheme 6): e.g., (i) with 1,4-dibromobutane (1.2 eq.) in the presence of LiHMDS (2.5 eq.) in THF [19]; (ii) with 1,2-dibromoethane (3.0 eq.) in the presence of butyllithium (BuLi, 4.0 eq.) and *N*,*N*-diisopropylethylamine (DIPEA) in THF [20]; (iii) with 1,2-bis(bromomethyl)-4-nitrobenzene (1.0 eq.) in the presence of BuLi (2.5 eq.) and tetramethylethylenediamine (TMEDA) in THF [21,22,23,24]; or (iv) with methyl 3,4-bis(bromomethyl)-benzoate (0.50 eq.) or *tert*-butyl 5,6-bis(chloromethyl)pyridine-3-carboxylate (0.60 eq.) in the presence of cesium carbonate (1.1 eq.) and sodium bromide in dimethyl formamide (DMF) [6,25,26].

All the above reactions led to the desired *C*(5)-alkyl or *C*(5)-dialkyl derivatives in low, moderate or undisclosed yields. The selectivity between the *C*(5) mono- and dialkyl products, and the formation of *N*(7)-alkylated by-products were not discussed.

**Scheme 7:**
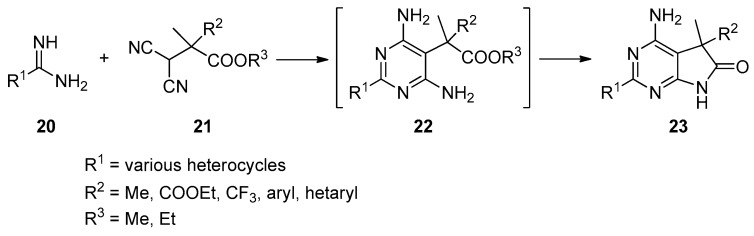
Examples for the ring-closure based approaches for the synthesis of *C*(5)-disubstituted 4-amino-1,3-diazaoxindoles **23**.

An alternative approach for the preparation of *N*(7)-unsubstituted-*C*(5)-mono- or *C*(5)-disubstituted 1,3-diazaoxindoles is also described [27,28,29,30,31]. Herein, the pyrrolo[2,3-*d*]pyrimidin-6-one scaffold is synthesized by a condensation of building blocks already containing the required substituents in the appropriate positions. This strategy is exemplified by the reaction sequence outlined in Scheme 7.

In the reaction of amidines **20** with the corresponding substituted malonitriles **21**, *C*(5)-disubstituted 1,3-diazaoxindoles **23** were obtained, obviously via pyrimidines **22**. The common drawback of these reactions is that the *C*(5) substituents of the target compounds have to be present in the starting materials **21**, thereby limiting the versatility of this approach.

## 2. Results and Discussion

**Scheme 8:**
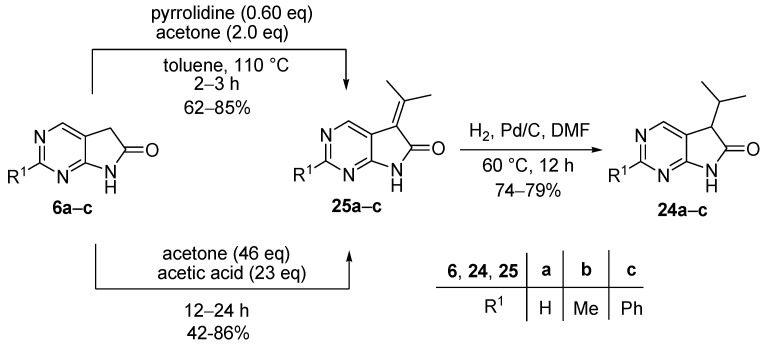
Two-step synthesis of 5-isopropyl-1,3-diazaoxindoles **24**.

In the present study, we aimed to elaborate a general procedure for the introduction of two different alkyl groups into the *C*(5) position of *N*(7)-unprotected 1,3-diazaoxindoles **6**. We decided to use the isopropyl group as the first alkyl substituent in position *C*(5), in order to increase steric hindrance in the second alkylation reaction. 5-Isopropyl-1,3-diazaoxindoles **24** can be prepared by using methods described in the literature for *C*(3)-alkylation of oxindoles [32,33,34]. Condensation of 1,3-diazaoxindoles **6** with acetone either under basic or acidic conditions afforded 5-isopropylidene-1,3-diazaoxindoles **25**, which were reduced by catalytic hydrogenation to give 5-isopropyl-1,3-diazaoxindoles **24** (Scheme 8).

**Scheme 9:**
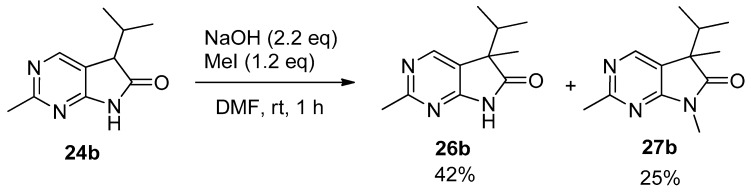
Formation of a product mixture during the alkylation of **24b** in the presence of NaOH.

In order to study the introduction of a second alkyl group into the *C*(5) position of 5-isopropyl-1,3-diazaoxindoles **24**, first we treated 2-methyl derivative **24b** with MeI (1.2 eq.) in the presence of sodium hydroxide (NaOH, 2.2 eq.) in DMF at room temperature (Scheme 9). In addition to *C*(5)-methylated product **26b**, a substantial amount of *C*(5),*N*(7)-dimethylated compound **27b** was also formed (besides unidentified minor products), as detected by the LC-MS analysis of the product mixture. After purification by flash chromatography, products **26b** and **27b** were isolated in 42 and 25% yields, respectively. This finding is in agreement with the experience gained with oxindoles [35,36,37,38]: sodium bases seem to be unsuitable for the high-yielding selective introduction of a second substituent into position *C*(5) also in the case of 1,3-diaza-derivatives **24**.

**Scheme 10:**
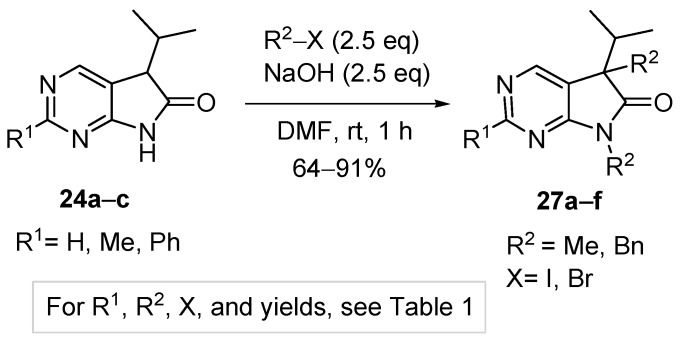
5,7-Dialkylation of 5-isopropyl-1,3-diazaoxindoles **24a**–**c** in the presence of NaOH.

Aiming at the elaboration of an efficient method for the synthesis of *C*(5),*N*(7)-disubstituted derivatives **27**, we carried out the alkylation of 2-unsubstituted (**24a**), 2-methyl- (**24b**) or 2-phenyl (**24c**) congeners with a higher excess of MeI (2.5 eq., Table 1, entries 1–3) or benzyl bromide (BnBr, 2.5 eq., entries 4–6) in the presence of NaOH (2.5 eq.) in DMF at ambient temperature. 5,7-Dimethyl(dibenzyl)-5-isopropyl-1,3-diazaoxindoles (**27a**–**f**) were obtained in good yields (Scheme 10).

As demonstrated by the reactions carried out using NaOH at our laboratory and by the procedures described in the literature using sodium, potassium or cesium bases for the deprotonation of 1,3-diazaoxindoles, selective alkylation at the *C*(5) position could not be achieved. Therefore, we continued our efforts by applying BuLi as the base in the alkylation reactions, which proved to be the optimal base in the analogous regioselective *C*(3)-alkylation reactions of *N*-unprotected oxindoles, particularly for the introduction of a second substituent into the carbonyl-adjacent *C*(3) position of *N*-unprotected 3-monoalkyloxindoles **9** (Scheme 2) [17,39].

**Scheme 11:**
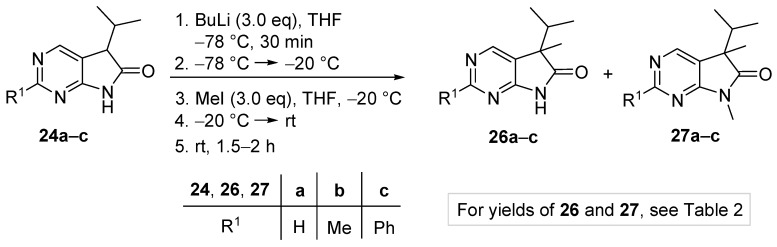
Methylation of 5-isopropyl-1,3-diazaoxindoles **24a**–**c** in the presence of a large excess of BuLi and MeI.

First we carried out reactions using a large excess of both BuLi and the alkylating agent in order to obtain a full picture of the regiochemistry of the alkylations. Methylation of compounds **24a**–**c** with BuLi (3.0 eq.) and MeI (3.0 eq.) was performed as follows (Scheme 11): (i) a solution of compounds **24a**–**c** in THF was added dropwise to the solution of BuLi in hexanes and THF −78 °C, and the reaction mixture was stirred at −78 °C for further 30 min; (ii) the reaction mixture was allowed to warm to −20 °C; (iii) a solution of MeI in THF was added dropwise at −20 °C; (iv) the mixture was allowed to warm to room temperature; (v) then it was stirred at this temperature for further 1.5–2 h. This protocol led to a mixture of 5-isopropyl-5-methyl- (compounds **26a**–**c**) and 5-isopropyl-5,7-dimethyl-1,3-diazaoxindoles **27a**–**c**. The yields of isolated products **26a**–**c** and **27a**–**c** after work-up and chromatographic purification can be found in Table 2 indicating that, despite the high excess of BuLi and MeI, the selectivity of the alkylation was quite high in the case of the 2-unsubstituted congener **24a** (entry 1), while it was poor in the case of the 2-methyl (**24b**) and 2-phenyl (**24c**) derivatives (entries 2–3).

**Figure 2:**
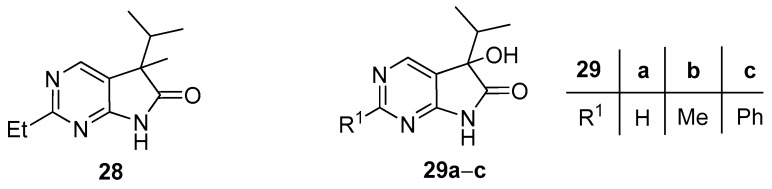
By-products of the methylation reactions of 5-isopropyl-1,3-diazaoxindoles **24**.

When deprotonation of 2-methyl derivative **24b** with BuLi (3.0 eq.) was performed at −20 °C (instead of −78 °C), and the reaction mixture was allowed to warm to room temperature, finally MeI (3.0 eq.) was added at this temperature (instead of −20 °C), methylation of the 2-methyl moiety was also observed resulting in 2-ethyl derivative **28** (Figure 2) as a minor product in 11% yield after flash chromatography, besides **26b** (30%) and **27b** (17%). A similar reactivity of the methyl moiety of 2-methylpyrimidines was observed by other research groups [40,41].

**Scheme 12:**
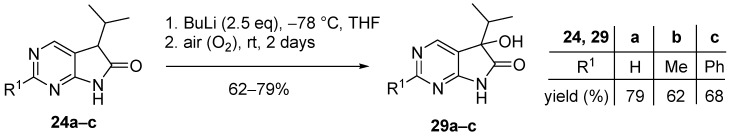
5-Hydroxylation of 5-isopropyl-1,3-diazaoxindoles **24a**–**c**.

As the consequence of an insufficient pre-inertization with argon, 5-hydroxy byproducts **29a**–**c** (Figure 2) were also isolated in some cases during our preliminary experiments. The targeted synthesis of the 5-hydroxy derivatives **29a**–**c** was carried out with BuLi (2.5 eq.) without alkylating agent and under non-inert conditions with good yields (Scheme 12). The presence of the hydroxy moiety in products **29a**–**c** renders further functionalizations possible thereby making these compounds valuable synthetic building blocks. An analogous oxidative side reaction via the corresponding hydroperoxide is also described in the oxindole family [42], and the corresponding 3-hydroxy derivatives in the oxindole series were also synthesised at our laboratory [17].

**Scheme 13:**
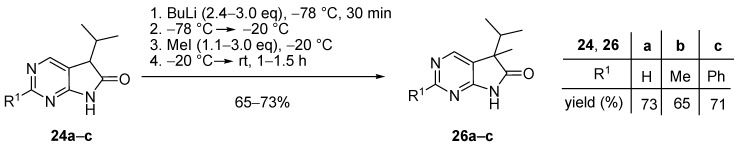
*C*(5)-Methylation of 5-isopropyl-1,3-diazaoxindoles **24a**–**c**.

In order to achieve full conversion of the starting materials **24**, to maximize the amount of monomethyl products **26** and to minimize that of dimethyl byproducts **27**, various conditions were explored, including the excess of BuLi and MeI, different deprotonation temperatures and the temperature of MeI addition (Scheme 13). It was found that significantly different protocols had to be used for starting materials **24a**, **24b** and **24c**. Deprotonation with BuLi leading to the formation of the corresponding dianions was performed at −78 °C in each case, although with different amounts of BuLi (2.4–3.0 eq.). Then, contrary to our earlier observations with 3-monoalkyloxindoles (**9**) where MeI was also added at −78 °C [17], for 1,3-diazaoxindoles **24a**–**c** we found that addition of MeI had to be carried out at −20 °C so that starting materials **24a**–**c** are fully consumed. Finally, the reaction mixture was allowed to warm to ambient temperature. When using 2-phenyl derivative **24c** as the starting material, a significant amount (19%) of 5,7-dimethyl derivative **27c** was formed even with 1.4 eq. of MeI, therefore the reaction was finally performed with a lower excess (1.1 eq.) of the alkylating agent. Using the optimized conditions, *C*(5)-methylated products **26a**–**c** were obtained in good yields (65–73%).

**Scheme 14:**
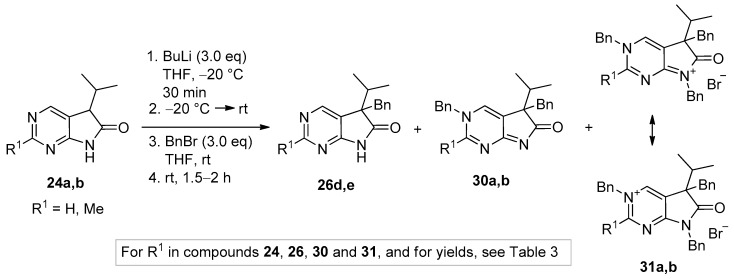
Benzylation of 5-isopropyl-1,3-diazaoxindoles **24a**,**b** in the presence of a large excess of BuLi and BnBr.

Next, alkylations with BnBr were attempted. When the deprotonation of 2-unsubstituted (**24a**) and 2-methyl-1,3-diazaoxindole (**24b**) with BuLi (3.0 eq.) was performed at −20 °C, then the addition of BnBr (3.0 eq.) at room temperature, surprising results were observed. After the work-up of the reaction mixture by flash chromatography, in addition to *C*(5)-benzylated derivatives **26d**,**e**, *N*(3),*C*(5)-dibenzylated compounds **30a**,**b** and *N*(3),*C*(5),*N*(7)-tribenzylated quaternary bromide salts **31a**,**b** were also isolated (Scheme 14), with a product distribution quite different in the two reactions (compare entries 1 and 2 in Table 3) and also substantially different from those of the alkylations performed with 3.0 eq. MeI (Scheme 11). It is also noteworthy that benzylation of the 2-methyl moiety of **24b** was not detected.

**Scheme 15:**
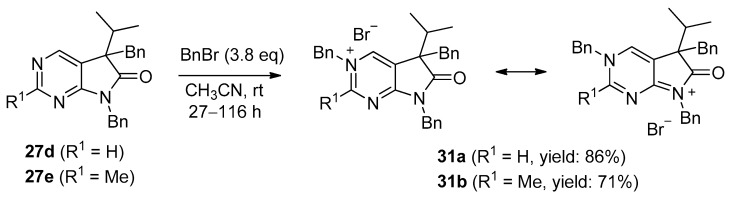
Targeted synthesis of 3,5,7-tribenzyl derivatives **31a**,**b** starting from 5,7-dibenzyl derivatives **27d**,**e**.

Tribenzylated quaternary bromide salts **31a**,**b** were also prepared by benzylation of 5,7-dibenzyl-1,3-diazaoxindoles **27d**,**e** with a high excess of BnBr (3.8 eq.) in acetonitrile at ambient temperature (Scheme 15). After long reaction times (27–116 h), quaternary salts **31a** and **31b** were obtained in good yields (86% and 71%, respectively).

**Figure 3:**
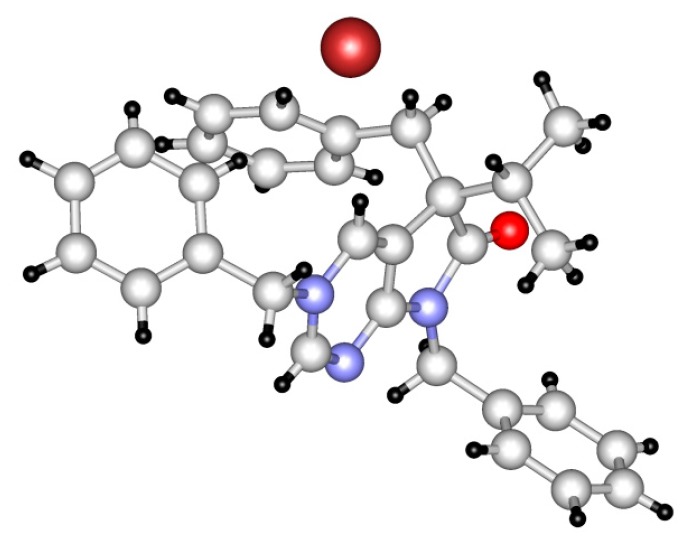
Perspective view of bromide salt **31a**.

**Figure 4:**
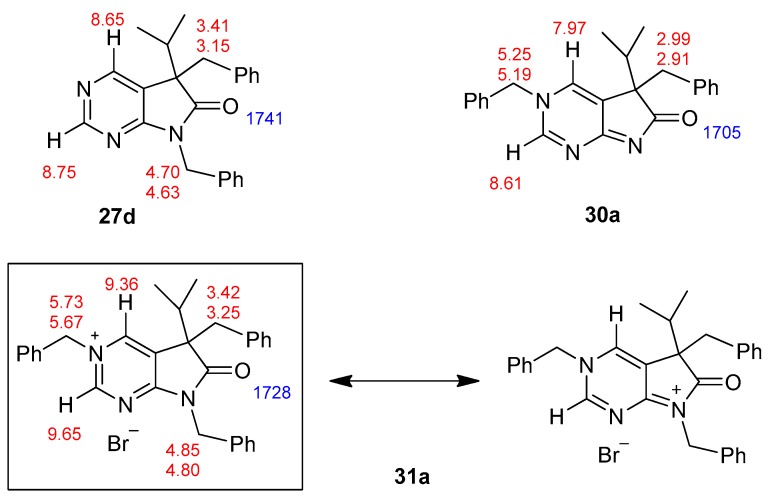
Characteristic ^1^H-NMR (red, ppm) and IR (blue, cm^−1^) signals of 5,7-dibenzyl- (**27d**), 3,5-dibenzyl- (**30a**) and 3,5,7-tribenzyl- (**31a**) derivatives.

The structure of tribenzyl derivative **31a** was also proven by single-crystal X-ray diffraction (Figure 3 and Supplementary Materials). A detailed analysis of the ^1^H-NMR and IR signals of **31a**, compared to those of 5,7-dibenzyl (**27d**) and 3,5-dibenzyl (**30a**) derivatives, showed that the electron distribution of **31a** is unambiguously closer to the framed resonance structure (Figure 4). The strong downfield shift of *C*(2)-H and *C*(4)-H signals in **31a** in comparison to **27d** and **30a** indicate that the positive charge is mainly located in the pyrimidine ring. The *N*(3)-CH_2_ signal of **31a** is also shifted by ca. 0.5 ppm when compared to the analogous signal in **30a**, due to the positive charge of the *N*(3) atom in **31a**, while the *N*(7)-CH_2_ signal remains practically unchanged when compared to **27d**. The presence of a positively charged nitrogen atom in the pyrrole ring (as it is the case in the resonance structure of **31a** on the right hand side) would shift the IR signal of the carbonyl moiety of **31a** to a frequency higher than the carbonyl signal detected in **27d** (1741 cm^−1^).

**Scheme 16:**
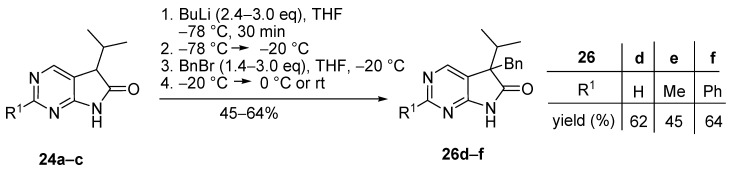
*C*(5)-Benzylation of 5-isopropyl-1,3-diazaoxindoles **24a**–**c**.

As the next step, the optimal conditions of *C*(5)-monobenzylation were sought. Similarly to the methylation reactions described above (Scheme 13), different protocols had to be elaborated for the benzylation of starting materials **24a**, **24b** and **24c** (Scheme 16). BnBr was added at −20 °C in an excess of 1.4–3.0 eq. Use of lower excesses led in each case to significant amounts of the starting materials in the crude product, probably due to the higher steric hindrance of BnBr compared to MeI. By using the optimized conditions, *C*(5)-benzylated products **26d**–**f** were obtained in acceptable yields (45–64%).

The different behavior of the 2-H (**24a**), 2-Me (**24b**) and 2-Ph (**24c**) derivatives observed in the *C*(5)-alkylation reactions can be attributed first of all to the different character of the *C*(2) substituents, on the other hand to the different alkylating agents, giving rise to various side reactions. Besides the 5,7-dialkylated 1,3-diazaoxindoles **27**, the formation of *C*(2)-ethyl derivative **28** and products **30a**,**b** and **31a**,**b** benzylated at the *N*(3) position was observed, indicating that the selective alkylation at the *C*(5) position of *N*(7)-unsubstituted 5-isopropyl-1,3-diazaoxindoles **24** is an even more challenging task than the analogous selective *C*(3)-alkylation of oxindoles.

## 3. Experimental Section

### 3.1. General Information

Compounds **24a**–**c**, **25a**–**c**, **26a**–**f**, **27a**–**f**, **28**, **29a**–**c**, **30a**,**b**, and **31a**,**b** are new and characterized below. All melting points were determined on an OptiMelt Automated Melting Point System by Stanford Research Systems (Sunnyvale, CA, USA). IR spectra were obtained on a Bruker ALPHA FT-IR spectrometer (Bruker, Billerica, MA, USA). NMR spectra were recorded either on a Bruker AVANCE III 400 (^1^H and ^13^C frequencies were 400 and 100 MHz, respectively) or a Bruker AVANCE III HD 600 (^1^H and ^13^C frequencies were 600 and 150 MHz, respectively) spectrometer. CDCl_3_, DMSO-*d*_6_ or TFA-*d* was used as the solvent and tetramethylsilane (TMS) as the internal standard. Chemical shifts (δ) and coupling constants (*J*) are given in ppm and in Hz, respectively. Some representatives of compound families 26–31 were also investigated using special NMR measurements (^1^H-^1^H COSY, ^1^H-^13^C HSQC, ^1^H-^13^C HMBC, selective NOESY). High-resolution mass spectra (HRMS spectra) were recorded either on a Micromass GCT (Waters, Milford, MA, USA) with EI (direct inlet) or on a Bruker Q-TOF MAXIS Impact mass spectrometer coupled to a Dionex Ultimate 3000 RS HPLC system (Thermo Fisher Scientific, Waltham, MA, USA) with a diode array detector. The reactions were followed by analytical thin layer chromatography on silica gel 60 F_254_ (Merck, Darmstadt, Germany) and by HPLC-MS on a Shimadzu LC-20 HPLC equipment (Kyoto, Japan) with an SPD-M20A diode-array detector coupled with a LCMS-2020 mass spectrometer. All unspecified reagents were purchased from commercial sources.

### 3.2. General Procedure I for the Synthesis of Compounds ***25a**–**c***

A mixture of the corresponding 1,3-diazaoxindole (**6a**, **6b** or **6c**), pyrrolidine (0.60 eq.) and acetone (2.0 eq.) in toluene was stirred at reflux temperature for 2–4 h. The water was removed with a Dean-Stark apparatus. After the reaction was complete, the volatile components were removed in vacuo at 60 °C and the residue was triturated thoroughly with hexane. The precipitate was filtered off, washed with hexane four times and dried.

### 3.3. General Procedure II for the Synthesis of Compounds ***25a**–**c***

A mixture of the corresponding 1,3-diazaoxindole (**6a**, **6b** or **6c**), acetic acid (24 eq.) and acetone (46 eq.) was stirred at reflux temperature under argon atmosphere (Ar) for 12–48 h. After the reaction was complete, the volatile components were removed in vacuo at 40 °C and the residue was triturated thoroughly with diethyl ether (DEE) or water. The precipitate was filtered off and dried.

*5-(1-Methylethylidene)-5*,*7-dihydro-6H-pyrrolo[2*,*3-d]pyrimidin-6-one* (**25a**). Method A: This compound was prepared according to General Procedure I using **6a** (500 mg, 3.7 mmol), pyrrolidine (0.18 mL, 0.16 g, 2.2 mmol), acetone (0.54 mL, 0.43 g, 7.4 mmol) in toluene (10 mL). The crude product was recrystallized from *N*,*N*-dimethylformamide (DMF) to give **25a** (399 mg, 62%) as pale brown crystals, m.p. 200 °C (decomp., DMF). IR (KBr) ν 1718 cm^−1^. ^1^H-NMR (400 MHz, DMSO-*d*_6_) δ 11.52 (br s, 1H), 8.66 (s, 1H), 8.63 (s, 1H), 2.53 (s, 3H), 2.36 (s, 3H). ^13^C-NMR (100 MHz, DMSO-*d*_6_) δ 167.6, 160.4_0_, 160.3_6_, 156.1, 147.3, 119.1, 116.5, 25.7, 22.7. HRMS calcd. for C_9_H_9_N_3_O [M˙]^+^ 175.0746; found 175.0762. Method B: This compound was also prepared according to General Procedure II using **6a** (3.0 g, 22.2 mmol), acetic acid (30 mL, 524 mmol), acetone (75 mL, 59.25 g, 1022 mmol). The crude product was recrystallized from DMF to give **25a** (3.34 g, 86%) as pale brown crystals. Spectral data were identical with those of the product obtained using General Procedure I.

*2-Methyl-5-(1-methylethylidene)-5*,*7-dihydro-6H-pyrrolo[2*,*3-d]pyrimidin-6-one* (**25b**). Method A: This compound was prepared according to General Procedure I using **6b** (15.00 g, 100 mmol), pyrrolidine (4.96 mL, 4.27 g, 60 mmol), acetone (14.68 mL, 11.60 g, 200 mmol) in toluene (150 mL). The crude product was recrystallized from DMF to give **25b** (16.08 g, 85%) as yellow crystals, m.p. 303 °C (decomp., DMF). IR (KBr) ν 1709, 1434 cm^−1^. ^1^H-NMR (600 MHz, TFA-*d*) δ 11.71 (br s, 1H), 8.74 (s, 1H), 2.99 (s, 3H), 2.85 (s, 3H), 2.62 (s, 3H). ^13^C-NMR (150 MHz, TFA-*d*) δ 179.8, 171.1, 166.1, 165.6, 138.2, 118.4, 118.1, 27.9, 25.9, 23.1. HRMS calcd. for C_10_H_11_N_3_O [M˙]^+^ 189.0902; found 189.0891. Method B: This compound was also prepared according to General Procedure II using **6b** (180 mg, 1.21 mmol), acetic acid (1.66 mL, 1.74 g, 29.04 mmol), acetone (4.09 mL, 3.23 g, 55.66 mmol). The product **25b** was obtained as yellow crystals (98 mg, 42%). Spectral data were identical with those of the product obtained using General Procedure I.

*2-Phenyl-5-(1-methylethylidene)-5*,*7-dihydro-6H-pyrrolo[2*,*3-d]pyrimidin-6-one* (**25c**). Method A: This compound was prepared according to General Procedure I using **6c** (2.00 g, 9.50 mmol), pyrrolidine (0.47 mL, 0.41 g, 5.7 mmol), acetone (1.39 mL, 1.10 g, 19 mmol) in toluene (20 mL). The crude product was recrystallized from toluene–DMF to give **25c** (1.74 g, 73%) as yellow crystals, m.p. 211 °C (decomp., toluene–DMF). IR (KBr) ν 1706, 1592 cm^−1^. ^1^H-NMR (400 MHz, DMSO-*d*_6_) δ 11.60 (br s, 1H), 8.78 (s, 1H), 8.37–8.34 (m, 2H), 7.52−7.50 (m, 3H), 2.54 (s, 3H), 2.38 (s, 3H). ^13^C-NMR (100 MHz, DMSO-*d*_6_) δ 168.0, 161.1, 160.9, 159.4, 147.7, 137.5, 130.8, 128.8, 127.7, 119.3, 114.8, 25.7, 22.6. HRMS calcd. for C_15_H_13_N_3_O [M˙]^+^ 251.1059; found 251.1056. Method B: This compound was also prepared according to General Procedure II using **6c** (120 mg, 0.57 mmol), acetic acid (0.78 mL, 0.82 g, 13.70 mmol), acetone (1.90 mL, 1.50 g, 26.20 mmol). The product **25c** was obtained as orange crystals (89 mg, 62%). Spectral data were identical with those of the product obtained using General Procedure I.

### 3.4. General Procedure III for the Synthesis of Compounds ***24a**–**c***

Diazaoxindole derivatives **25a**–**c** were hydrogenated at 60 °C under atmospheric pressure in DMF using activated palladium on charcoal (Pd/C, 10% Pd) catalyst for 12 h. After the reaction was completed, the catalyst was filtered off and washed with DMF three times. The filtrate and the washings were combined and the solvent was removed at 60 °C under reduced pressure. The residue was triturated thoroughly with water, the precipitate was filtered off and dried.

*5-(1-Methylethyl)-5*,*7-dihydro-6H-pyrrolo[2*,*3-d]pyrimidin-6-one* (**24a**). This compound was prepared according to General Procedure III using **25a** (500 mg, 2.85 mmol) and Pd/C catalyst (140 mg) in DMF (50 mL). The crude product was recrystallized from toluene–DMF to give **25a** (389 mg, 77%) as colorless crystals, m.p. 127–128 °C (toluene–DMF). IR (KBr) ν 1739, 1596 cm^−1^. ^1^H-NMR (600 MHz, CDCl_3_) δ 10.98 (br s, 1H), 8.87 (d, *J* = 0.8 Hz, 1H), 8.46 (d, *J* = 0.8 Hz, 1H), 3.57 (d, *J* = 4.0 Hz, 1H), 2.78 (m, 1H), 1.22 (d, *J* = 6.8 Hz, 3H), 0.92 (d, *J* = 6.8 Hz, 3H). ^13^C-NMR (150 MHz, CDCl_3_) δ 177.6, 164.3, 157.0, 149.3, 120.3, 50.3, 30.1, 20.2, 17.6. HRMS calcd. for C_9_H_11_N_3_O [M˙]^+^ 177.0902; found 177.0910.

*2-Methyl-5-(1-methylethyl)-5*,*7-dihydro-6H-pyrrolo[2*,*3-d]pyrimidin-6-one* (**24b**). This compound was prepared according to General Procedure III using **25b** (18.2 g, 96 mmol) and Pd/C catalyst (3.0 g) in DMF (300 mL). The crude product was recrystallized from acetonitrile to give **24b** (13.57 g, 74%) as colorless crystals, m.p. 180–181 °C (acetonitrile). IR (KBr) ν 1738, 1618 cm^−1^. ^1^H-NMR (400 MHz, CDCl_3_) δ 10.84 (br s, 1H), 8.31 (s, 1H), 3.49 (d, *J* = 4.0 Hz, 1H), 2.75 (s, 3H), 2.54 (m, 1H), 1.20 (d, *J* = 6.8 Hz, 3H), 0.91 (d, *J* = 6.8 Hz, 3H). ^13^C-NMR (100 MHz, CDCl_3_) δ 177.6, 167.0, 164.5, 149.7, 117.0, 50.2, 30.1, 25.5, 20.4, 17.7. HRMS calcd. for C_10_H_13_N_3_O [M˙]^+^ 191.1059; found 191.1070.

*2-Phenyl-5-(1-methylethyl)-5*,*7-dihydro-6H-pyrrolo[2*,*3-d]pyrimidin-6-one* (**24c**). This compound was prepared according to General Procedure III using **25c** (17.3 g, 68.80 mmol) and Pd/C catalyst (3.0 g) in DMF (250 mL). The crude product was recrystallized from toluene to give **24c** (13.77 g, 79%) as colorless crystals, m.p. 178–180 °C (toluene). IR (KBr) ν 1738, 1620 cm^−1^. ^1^H-NMR (400 MHz, DMSO-*d*_6_) δ 11.56 (br s, 1H), 8.54 (s, 1H), 8.35–8.33 (m, 2H), 7.53–7.51 (m, 3H), 3.66 (d, *J* = 3.7 Hz, 1H), 2.40 (m, 1H), 1.10 (d, *J* = 6.8 Hz, 3H), 0.83 (d, *J* = 6.8 Hz, 3H). ^13^C-NMR (100 MHz, DMSO-*d*_6_) δ 178.0, 165.4, 162.3, 149.7, 137.5, 130.8, 128.8, 127.7, 118.6, 49.7, 29.6, 20.0, 18.0. HRMS calcd. for C_15_H_15_N_3_O [M˙]^+^ 253.1215; found 253.1220.

*5-Methyl-5-(1-methylethyl)-5*,*7-dihydro-6H-pyrrolo[2*,*3-d]pyrimidin-6-one* (**26a**). To a mixture of BuLi in hexane (5.63 mL, 9.00 mmol, 3.0 eq., 1.6 M) and THF (8 mL), the solution of 5-(1-methylethyl)-5,7-dihydro-6*H*-pyrrolo[2,3-*d*]pyrimidin-6-one (**24a**, 532 mg, 3.00 mmol) in THF (10 mL) was added dropwise at −78 °C under Ar atmosphere. The mixture was stirred for 30 min, then the temperature was allowed to warm to –20 °C and MeI (0.56 mL, 1.28 g, 9.00 mmol, 3.00 eq.) in THF (1 mL) was added dropwise. The acetone-dry ice bath was removed and the stirring was continued for further 1 h. The mixture was quenched with saturated ammonium chloride (NH_4_Cl) solution (10 mL), water (5 mL) was added, then the mixture was stirred for 15 min under Ar atmosphere. The layers were separated and the aqueous layer was extracted with ethyl acetate (EtOAc, 3 × 15 mL). The combined organic layer was washed with brine (30 mL), dried over anhydrous MgSO_4_, filtered and the solvent was removed in vacuo at 40 °C. The residue crystallized upon treatment with hexane (5 mL), then it was filtered off, washed with diisopropyl ether (DIPE, 2 × 2 mL) and dried. The product **26a** was obtained as yellow crystals (420 mg, 73%), m.p. 149–151 °C (hexane–EtOAc, colorless crystals). IR (KBr) ν 1732, 1603 cm^−1^. ^1^H-NMR (600 MHz, CDCl_3_) δ 9.85 (br s, 1H), 8.83 (s, 1H), 8.36 (s, 1H), 2.21 (sp, *J* = 6.9 Hz, 1H), 1.47 (s, 3H), 1.11 (d, *J* = 6.9 Hz, 3H), 0.81 (d, *J* = 6.9 Hz, 3H). ^13^C-NMR (150 MHz, CDCl_3_) δ 181.0, 163.0, 157.0, 149.1, 125.1, 51.6, 34.9, 20.9, 17.7, 17.2. HSQC: 8.83–157.0; 8.36–149.1; 2.21–34.9; 1.47–20.9; 1.11–17.2; 0.81–17.7. HMBC (*J*_long-range_ = 8 Hz; characteristic cross-peaks): 8.83–163.0, 149.1; 8.36–163.0, 157.0, 125.1; 2.21–125.1, 51.6, 17.7, 17.2; 1.47–181.0, 125.1, 51.6, 34.9; 1.11–51.6, 34.9, 17.7; 0.81–51.6, 34.9, 17.2. Selective NOESY: 1.47–8.36, 2.21, 1.11, 0.81; 1.11–8.36, 2.21, 1.47, 0.81; 0.81–8.36, 2.21, 1.47, 1.11. HRMS calcd. for C_10_H_13_N_3_O [M˙]^+^ 191.1059; found 191.1075. 

*2*,*5-Dimethyl-5-(1-methylethyl)-5*,*7-dihydro-6H-pyrrolo[2*,*3-d]pyrimidin-6-one* (**26b**). To a mixture of BuLi in hexane (4.50 mL, 7.20 mmol, 2.4 eq., 1.6 M) and THF (5 mL), the solution of 2-methyl-5-(1-methylethyl)-5,7-dihydro-6*H*-pyrrolo[2,3-*d*]pyrimidin-6-one (**24b**, 574 mg, 3.00 mmol) in THF (15 mL) was added dropwise at −78 °C under Ar atmosphere. The mixture was stirred for 30 min, then the temperature was allowed to warm to –20 °C and MeI (0.26 mL, 0.60 g, 4.20 mmol, 1.4 eq.) in THF (1 mL) was added dropwise. The acetone-dry ice bath was removed and the reaction mixture was allowed to warm to room temperature. The stirring was continued for further 1.5 h. The mixture was quenched with saturated NH_4_Cl solution (10 mL), water (5 mL) was added, then the mixture was stirred for 15 min under Ar atmosphere. The layers were separated and the aqueous layer was extracted with EtOAc (3 × 15 mL). The combined organic layer was washed with brine (30 mL), dried over anhydrous MgSO_4_, filtered and the solvent was removed in vacuo at 40 °C. The residue crystallized upon treatment with hexane (5 mL), then it was filtered off, washed with DIPE (2 × 2 mL) and dried. The product **26b** was obtained as yellow crystals (399 mg, 65%), m.p. 190–191 °C (hexane–EtOAc, colorless crystals). IR (KBr) ν 1744, 1621, 1429, 1190 cm^−1^. ^1^H-NMR (600 MHz, CDCl_3_) δ 11.36 (br s, 1H), 8.30 (s, 1H), 2.49 (s, 3H), 2.01 (sp, *J* = 6.8 Hz, 1H), 1.30 (s, 3H), 0.98 (d, *J* = 6.8 Hz, 3H), 0.66 (d, *J* = 6.8 Hz, 3H). ^13^C-NMR (150 MHz, CDCl_3_) δ 181.5, 166.1, 163.9, 149.0, 121.4, 50.6, 34.3, 25.8, 20.7, 17.6, 17.2. HRMS calcd. for C_11_H_15_N_3_O [M˙]^+^ 205.1215; found 205.1219.

*5-Methyl-2-phenyl-5-(1-methylethyl)-5*,*7-dihydro-6H-pyrrolo[2*,*3-d]pyrimidin-6-one* (**26c**). To a mixture of BuLi in hexane (2.96 mL, 4.73 mmol, 2.4 eq., 1.6 M) and THF (3 mL), the solution of 2-phenyl-5-(1-methylethyl)-5,7-dihydro-6*H*-pyrrolo[2,3-*d*]pyrimidin-6-one (**24c**, 500 mg, 1.97 mmol) in THF (8 mL) was added dropwise at −78 °C under Ar atmosphere. The mixture was stirred for 30 min, then the temperature was allowed to warm to −20 °C, and MeI (0.13 mL, 0.31 g, 2.17 mmol, 1.1 eq.) in THF (1 mL) was added dropwise. The acetone-dry ice bath was removed and the reaction mixture was stirred for further 1 h. The mixture was quenched with saturated NH_4_Cl solution (10 mL), water (5 mL) was added, then the mixture was stirred for 15 min under Ar atmosphere. The layers were separated and the aqueous layer was extracted with EtOAc (3 × 15 mL). The combined organic layer was washed with brine (30 mL), dried over anhydrous MgSO_4_, filtered and the solvent was removed in vacuo at 40 °C. The residue crystallized upon treatment with hexane (5 mL), then it was filtered off, washed with DIPE (2 × 2 mL) and dried. The product **26c** was purified by gradient elution column chromatography using hexane and EtOAc as the eluents. The product **26c** was triturated in hexane (3 mL), washed with DIPE (2 × 1 mL) and dried to give colorless crystals (372 mg, 71%), m.p. 155–156 °C. IR (KBr) ν 1728, 1599, 1405, 1201 cm^−1^. ^1^H-NMR (600 MHz, CDCl_3_) δ 8.58 (br s, 1H), 8.43 (s, 1H), 8.37–8.35 (m, 2H), 7.49–7.48 (m, 3H), 2.23 (sp, *J* = 6.8 Hz, 1H), 1.49 (s, 3H), 1.13 (d, *J* = 6.8 Hz, 3H), 0.83 (d, *J* = 6.8 Hz, 3H). ^13^C-NMR (150 MHz, CDCl_3_) δ 181.1, 163.7, 163.0, 149.5, 137.1, 130.9, 128.6, 128.1, 122.3, 51.7, 35.0, 21.0, 17.8, 17.3. HRMS calcd. for C_16_H_17_N_3_O [M˙]^+^ 267.1372; found 267.1370.

*5-Benzyl-5-(1-methylethyl)-5*,*7-dihydro-6H-pyrrolo[2*,*3-d]pyrimidin-6-one* (**26d**). To a mixture of BuLi in hexane (2.81 mL, 4.50 mmol, 3.0 eq., 1.6 M) and THF (3 mL), the solution of 5-(1-methylethyl)-5,7-dihydro-6*H*-pyrrolo[2,3-*d*]pyrimidin-6-one (**24a**, 266 mg, 1.50 mmol) in THF (5 mL) was added dropwise at −78 °C under Ar atmosphere. The mixture was stirred for 30 min, then the temperature was allowed to warm to −20 °C and BnBr (0.53 mL, 0.77 g, 4.50 mmol, 3.0 eq.) in THF (1 mL) was added dropwise. The acetone-dry ice bath was removed and the reaction mixture was stirred for further 1 h. The mixture was quenched with saturated NH_4_Cl solution (10 mL), water (5 mL) was added, then the mixture was stirred for 15 min under Ar atmosphere. The layers were separated and the aqueous layer was extracted with EtOAc (3 × 15 mL). The combined organic layer was washed with brine (30 mL), dried over anhydrous MgSO_4_, filtered and the solvent was removed in vacuo at 40 °C. The residue crystallized upon treatment with hexane (5 mL), then it was filtered off, washed with DIPE (2 × 2 mL) and dried. The product **26d** was purified by gradient elution column chromatography using hexane and EtOAc as the eluents. The product **26d** was triturated in hexane (3 mL), washed with DIPE (2 × 1 mL) and dried to give **26d** (250 mg, 62%) as colorless crystals, m.p. 195–197 °C. IR (KBr) ν 1731, 1601 cm^−1^. ^1^H-NMR (600 MHz, CDCl_3_) δ 8.70 (br, 1H), 8.65 (s, 1H), 8.43 (s, 1H), 7.09–7.04 (m, 3H), 6.87 (m, 2H), 3.26 (d, *J* = 13.2 Hz, 1H), 3.21 (d, *J* = 13.2 Hz, 1H), 2.38 (sp, *J* = 6.8 Hz, 1H), 1.19 (d, *J* = 6.8 Hz, 3H), 0.86 (d, *J* = 6.8 Hz, 3H). ^13^C-NMR (150 MHz, CDCl_3_) δ 179.1, 163.0, 157.1, 149.8, 134.9, 129.7, 128.1, 127.0, 122.5, 58.5, 40.9, 35.0, 17.9, 17.6. HSQC: 8.65–157.1; 8.43–149.8, (7.09–7.04)–127.0, 128.1; 6.87–129.7; 3.26–40.9; 3.21–40.9; 2.38–35.0; 1.19–17.6; 0.86–17.9. HMBC (*J*_long-range_ = 7 Hz; characteristic cross-peaks): 8.65–163.0, 149.8; 8.43–163.0, 157.1, 122.5; (7.09–7.04)–134.9; 6.87–40.9; 3.26–135.0, 129.7, 58.5; 3.21–179.1, 135.0, 129.7, 58.5; 2.38–179.1, 122.5, 58.5, 17.9, 17.6; 1.19–58.5, 35.0, 17.9; 0.86–58.5, 35.0, 17.6. HRMS calcd. for C_16_H_17_N_3_O [M˙]^+^ 267.1372; found 267.1371.

*5-Benzyl-2-methyl-5-(1-methylethyl)-5*,*7-dihydro-6H-pyrrolo[2*,*3-d]pyrimidin-6-one* (**26e**). To a mixture of BuLi in hexane (2.36 mL, 3.66 mmol, 2.4 eq., 1.6 M) and THF (3 mL), the solution of 2-methyl-5-(1-methylethyl)-5,7-dihydro-6*H*-pyrrolo[2,3-*d*]pyrimidin-6-one (**24a**, 300 mg, 1.57 mmol) in THF (7 mL) was added dropwise at −78 °C under Ar atmosphere. The mixture was stirred for 30 min, then the temperature was allowed to warm to 0 °C and BnBr (0.26 mL, 0.38 g, 2.20 mmol, 1.4 eq.) in THF (1 mL) was added dropwise. The reaction mixture was allowed to warm to room temperature and stirred for further 3 h. The mixture was quenched with saturated NH_4_Cl solution (10 mL), water (5 mL) was added, then the mixture was stirred for 15 min under Ar atmosphere. The layers were separated and the aqueous layer was extracted with EtOAc (3 × 15 mL). The combined organic layer was washed with brine (30 mL), dried over anhydrous MgSO_4_, filtered and the solvent was removed in vacuo at 40 °C. The residue crystallized upon treatment with hexane (5 mL), it was filtered off, washed with hexane (2 × 2 mL) and DIPE (2 mL), then dried. The crude product **26e** was purified by gradient elution column chromatography using hexane and EtOAc as the eluents. This product **26e** was triturated in hexane (3 mL), washed with DIPE (2 × 1 mL) and dried to give **26e** (199 mg, 45%) as colorless crystals, m.p. 185–187 °C. IR (KBr) ν 1740, 1620, 1426 cm^−1^. ^1^H-NMR (400 MHz, CDCl_3_) δ 9.79 (br s, 1H), 8.29 (s, 1H), 7.07–7.05 (m, 3H), 6.90 (m, 2H), 3.23 (d, *J* = 13.2 Hz, 1H), 3.19 (d, *J* = 13.2 Hz, 1H), 2.60 (s, 3H), 2.34 (sp, *J* = 6.8 Hz, 1H), 1.17 (d, *J* = 6.8 Hz, 3H), 0.85 (d, *J* = 6.8 Hz, 3H). ^13^C-NMR (100 MHz, CDCl_3_) δ 179.7, 166.8, 163.6, 149.8, 135.3, 129.8, 128.0, 126.8, 119.2, 58.1, 40.8, 34.9, 25.5, 17.9, 17.6. HRMS calcd. for C_17_H_19_N_3_O [M˙]^+^ 281.1528; found 281.1524.

*5-Benzyl-2-phenyl-5-(1-methylethyl)-5*,*7-dihydro-6H-pyrrolo[2*,*3-d]pyrimidin-6-one* (**26f**). To a mixture of BuLi in hexane (1.89 mL, 4.73 mmol, 2.4 eq., 2.5 M) and THF (3 mL), the solution of 2-phenyl-5-(1-methylethyl)-5,7-dihydro-6*H*-pyrrolo[2,3-*d*]pyrimidin-6-one (**24c**, 500 mg, 1.97 mmol) in THF (6 mL) was added dropwise at −78 °C under Ar atmosphere. The mixture was stirred for 30 min, then the temperature was allowed to warm to –20 °C and BnBr (0.70 mL, 1.01 g, 5.91 mmol, 3.0 eq.) in THF (1 mL) was added dropwise. The acetone-dry ice bath was removed and the reaction mixture was allowed to warm to 0 °C. The stirring was continued for further 5 h at 0 °C. The mixture was quenched with saturated NH_4_Cl solution (10 mL), water (5 mL) was added, then the mixture was stirred for 15 min under Ar atmosphere. The layers were separated and the aqueous layer was extracted with EtOAc (3 × 15 mL). The combined organic layer was washed with brine (30 mL), dried over anhydrous MgSO_4_, filtered and the solvent was removed in vacuo at 40 °C. The residue crystallized upon treatment with hexane/DIPE = 3/1 (5 mL), then it was filtered off, washed with hexane/DIPE = 3/1 (2 × 2 mL) and dried. The product **26f** was obtained as yellow crystals (435 mg, 64%), m.p. 192–193 °C (hexane–EtOAc, colorless crystals). IR (KBr) ν 1728, 1625, 1410 cm^−1^. ^1^H-NMR (400 MHz, CDCl_3_) δ 8.52 (s, 1H), 8.31–8.28 (m, 2H), 7.96 (br s, 1H), 7.46–7.44 (m, 3H), 7.07–7.05 (m, 3H), 6.95–6.93 (m, 2H), 3.27 (s, 2H), 2.40 (sp, *J* = 6.9 Hz, 1H), 1.23 (d, *J* = 6.9 Hz, 3H), 0.88 (d, *J* = 6.9 Hz, 3H). ^13^C-NMR (100 MHz, CDCl_3_) δ 179.3, 163.5, 163.2, 150.1, 137.0, 135.2, 130.9, 129.8, 128.6, 128.1, 128.0, 126.9, 119.8, 58.5, 41.0, 35.2, 18.0, 17.7. HRMS calcd. for C_22_H_21_N_3_O [M˙]^+^ 343.1685; found 343.1660.

### 3.5. General Procedure IV for the Synthesis of Compounds ***27a**–**f***

To a mixture of NaOH (2.5 eq.) in DMF, the solution of 5-isopropyl-1,3-diazaoxindoles **24a**–**c** in DMF was added dropwise at room temperature, under Ar atmosphere and it was stirred for 30 min. Then the solution of the appropriate alkyl halide (2.5 eq.) in DMF was added dropwise at 15–20 °C. The stirring was continued for further 1 h. The reaction mixture was poured onto ice–water. The precipitate was filtered off or the aqueous layer was extracted with dichloromethane (DCM, 3 × 15 mL). The combined organic layer was washed with brine (5 × 50 mL), dried over anhydrous MgSO_4_, filtered and the solvent was removed in vacuo at 40 °C. Purification of the crude products was performed as described below.

*5*,*7-Dimethyl-5-(1-methylethyl)-5*,*7-dihydro-6H-pyrrolo[2*,*3-d]pyrimidin-6-one* (**27a**). This compound was prepared according to General Procedure IV using **24a** (275 mg, 1.55 mmol) in DMF (4 mL), NaOH (155 mg, 3.88 mmol) in DMF (2 mL) and MeI (0.24 mL, 0.55 g, 3.88 mmol) in DMF (1 mL). The residual oil was purified by gradient elution column chromatography using hexane and EtOAc as the eluents. The product was triturated in hexane (3 mL), washed with DIPE (2 × 1 mL) and dried to give **27a** (203 mg, 64%) as colorless crystals, m.p. 64–66 °C. IR (KBr) ν 1731, 1586, 1492 cm^−1^. ^1^H-NMR (600 MHz, CDCl_3_) δ 8.81 (s, 1H), 8.29 (s, 1H), 3.28 (s, 3H), 2.20 (sp, *J* = 7.0 Hz, 1H), 1.43 (s, 3H), 1.07 (d, *J* = 7.0 Hz, 3H), 0.71 (d, *J* = 7.0 Hz, 3H). ^13^C-NMR (150 MHz, CDCl_3_) δ 180.2, 163.7, 157.5, 148.1, 124.5, 50.9, 35.0, 25.1, 20.8, 17.6, 17.3. HSQC: 8.81–157.5; 8.29–148.1; 3.28–25.1; 2.20–35.0; 1.43–20.8; 1.07–17.3; 0.71–17.6. HMBC (*J*_long-range_ = 8 Hz; characteristic cross-peaks): 8.81–163.7, 148.1; 8.29–163.7, 157.5, 124.5; 3.28–180.2, 163.7; 1.43–180.2, 124.5, 50.9, 35.0; 1.07–50.9, 35.0, 17.6; 0.71–50.9, 35.0, 17.3. Selective NOESY: 1.43–8.29, 3.28, 2.20, 1.07, 0.71. HRMS calcd. for C_11_H_15_N_3_O [M˙]^+^ 205.1215; found 205.1223.

*2*,*5*,*7-Trimethyl-5-(1-methylethyl)-5*,*7-dihydro-6H-pyrrolo[2*,*3-d]pyrimidin-6-one* (**27b**). This compound was prepared according to General Procedure IV using **24b** (574 mg, 3.00 mmol) in DMF (7 mL), NaOH (300 mg, 7.5 mmol) in DMF (2 mL) and MeI (0.47 mL, 1.06 g, 7.5 mmol) in DMF (1 mL). The residual oil was purified by gradient elution column chromatography using hexane and EtOAc as the eluents to give **27b** (527 mg, 80%) as a colorless oil. IR (film) ν 1732, 1600, 1488 cm^−1^. ^1^H-NMR (400 MHz, CDCl_3_) δ 8.17 (s, 1H), 3.26 (s, 3H), 2.67 (s, 3H), 2.17 (sp, *J* = 6.9 Hz, 1H), 1.40 (s, 3H), 1.10 (d, *J* = 6.9 Hz, 3H), 0.69 (d, *J* = 6.9 Hz, 3H). ^13^C-NMR (100 MHz, CDCl_3_) δ 180.6, 167.1, 164.0, 148.0, 121.1, 50.7, 34.9, 26.1, 25.1, 20.9, 17.6, 17.3. HRMS calcd. for C_12_H_17_N_3_O [M˙]^+^ 219.1372; found 219.1373.

*5*,*7-Dimethyl-2-phenyl-5-(1-methylethyl)-5*,*7-dihydro-6H-pyrrolo[2*,*3-d]pyrimidin-6-one* (**27c**). This compound was prepared according to General Procedure IV using **24c** (507 mg, 2 mmol) in DMF (7 mL), NaOH (200 mg, 5.00 mmol) in DMF (2 mL) and MeI (0.31 mL, 0.71 g) in DMF (1 mL). The precipitate was filtered off, washed with water (3 × 5 mL) and dried. The product **27c** was obtained as colorless crystals (512 mg, 91%). An analytical sample was obtained by recrystallization from hexane, m.p. 106–107 °C (hexane). IR (KBr) ν 1716, 1678 cm^−1^. ^1^H-NMR (400 MHz, CDCl_3_) δ 8.48–8.45 (m, 2H), 8.36 (s, 1H), 7.50–7.49 (m, 3H), 3.36 (s, 3H), 2.21 (sp, *J* = 6.8 Hz, 1H), 1.45 (s, 3H), 1.10 (d, *J* = 6.8 Hz, 3H), 0.73 (d, *J* = 6.8 Hz, 3H). ^13^C-NMR (100 MHz, CDCl_3_) δ 180.7, 164.2, 163.6, 148.5, 137.4, 130.8, 128.5, 128.1, 122.1, 51.0, 35.1, 25.2, 20.9, 17.7, 17.3. HRMS calcd. for C_17_H_19_N_3_O [M˙]^+^ 281.1528; found 281.1522.

*5*,*7-Dibenzyl-5-(1-methylethyl)-5*,*7-dihydro-6H-pyrrolo[2*,*3-d]pyrimidin-6-one* (**27d**). This compound was prepared according to General Procedure IV using **24a** (250 mg, 1.41 mmol) in DMF (2 mL), NaOH (141 mg, 3.53 mmol) in DMF (2 mL) and BnBr (0.42 mL, 0.60 g, 3.53 mmol) in DMF (1 mL). The residual oil was purified by gradient elution column chromatography using hexane and EtOAc as the eluents. The product was triturated in hexane (3 mL), washed with DIPE (2 × 1 mL) and dried to give **27d** (353 mg, 70%) as colorless crystals, m.p. 65–66 °C. IR (KBr) ν 1741, 1496 cm^−1^. ^1^H-NMR (600 MHz, DMSO-*d*_6_) δ 8.75 (s, 1H), 8.65 (s, 1H), 7.20–7.16 (m, 3H), 7.07 (m, 1H), 6.99 (t, *J* = 7.6 Hz, 2H), 6.83 (m, 2H), 6.81 (m, 2H), 4.70 (d, *J* = 15.4 Hz, 1H), 4.63 (d, *J* = 15.4 Hz, 1H), 3.41 (d, *J* = 13.0 Hz, 1H), 3.15 (d, *J* = 13.0 Hz, 1H), 2.30 (sp, *J* = 6.8 Hz, 1H), 1.08 (d, *J* = 6.8 Hz, 3H), 0.68 (d, *J* = 6.8 Hz, 3H). ^13^C-NMR (150 MHz, DMSO-*d*_6_) δ 178.2, 163.1, 157.4, 150.3, 135.8, 135.7, 129.8, 128.5, 128.0, 127.4, 127.2, 126.8, 121.6, 57.3, 41.8, 39.7, 35.2, 17.7, 17.6. COSY: (7.20–7.16)–6.83; 6.99–6.81; 3.41–3.15; 2.30–1.08, 0.68. HSQC: 8.75–150.3; 8.65–157.4; (7.20–7.16)–128.5, 127.4; 7.07–126.8; 6.99–128.0; 6.83–127.2; 6.81–129.8; 4.70–41.8; 4.63–41.8; 3.41–39.7; 3.15–39.7; 2.30–35.2; 1.08–17.7; 0.68–17.6. HMBC (*J*_long-range_ = 7 Hz; characteristic cross-peaks): 8.75–163.1, 157.4, 121.6; 6.83–41.8; 6.81–39.7; (7.20–7.16)–135.7; 6.99–135.8; 4.70–178.2, 163.1, 135.7, 127.2; 4.63–178.2, 163.1, 135.7, 127.2; 3.41–178.2, 135.8, 129.8, 57.3; 3.15–135.8, 129.8, 121.6, 57.3; 2.30–121.6, 57.3, 17.7, 17.6; 1.08–57.3, 35.2; 0.68–57.3, 35.2. COSY: (7.20–7.16)–6.83; 6.99–6.81; 3.41–3.15; 2.30–1.08, 0.68. HRMS calcd. for C_23_H_23_N_3_O [M˙]^+^ 357.1841; found 357.1829.

*5*,*7-Dibenzyl-2-methyl-5-(1-methylethyl)-5*,*7-dihydro-6H-pyrrolo[2*,*3-d]pyrimidin-6-one* (**27e**). This compound was prepared according to General Procedure IV using **24b** (200 mg, 1.05 mmol) in DMF (2 mL), NaOH (105 mg, 2.63 mmol) in DMF (2 mL) and BnBr (0.31 mL, 0.45 g, 2.63 mmol) in DMF (1 mL). The precipitate was filtered off, washed with water (3 × 5 mL) and dried. The product **27e** was obtained as orange crystals (320 mg, 82%). An analytical sample was obtained by recrystallization from a mixture of hexane and EtOAc to give **27e** as colorless crystals, m.p. 84–86 °C (hexane–EtOAc). IR (KBr) ν 1736, 1478 cm^−1^. ^1^H-NMR (400 MHz, DMSO-*d*_6_) δ 8.61 (br s, 1H), 7.21–7.17 (m, 3H), 7.07 (t, *J* = 7.4 Hz, 1H), 7.00 (t, *J* = 7.4 Hz, 2H), 6.86–6.80 (m, 4H), 4.67 (d, *J* = 15.4 Hz, 1H), 4.61 (d, *J* = 15.4 Hz, 1H), 3.37 (d, *J* = 13.0 Hz, 1H), 3.12 (d, *J* = 13.0 Hz, 1H), 2.44 (s, 3H), 2.26 (sp, *J* = 6.8 Hz, 1H), 1.07 (d, *J* = 6.8 Hz, 3H), 0.64 (d, *J* = 6.8 Hz, 3H). ^13^C-NMR (100 MHz, DMSO-*d*_6_) δ 178.5, 166.3, 163.4, 150.2, 135.9_0_, 135.8_5_, 129.8, 128.5, 128.0, 127.3, 127.1, 126.7, 118.1, 57.0, 41.7, 39.6, 35.2, 25.9, 17.6_04_, 17.5_96_. HRMS calcd. for C_24_H_25_N_3_O [M˙]^+^ 371.1998; found 371.1989.

*5*,*7-Dibenzyl-5-(1-methylethyl)-2-phenyl-5*,*7-dihydro-6H-pyrrolo[2*,*3-d]pyrimidin-6-one* (**27f**). This compound was prepared according to General Procedure IV using **24c** (760 mg, 3.00 mmol) in DMF (10 mL), NaOH (300 mg, 7.50 mmol) in DMF (2 mL) and BnBr (0.89 mL, 1.28 g, 7.50 mmol) in DMF (2 mL). The residual oil was purified by gradient elution column chromatography using hexane and EtOAc as the eluents to give **27f** (1062 mg, 82%) as a colorless oil. IR (film) ν 1732, 1596, 1475, 1398 cm^−1^. ^1^H-NMR (600 MHz, CDCl_3_) δ 8.49 (s, 1H), 8.38 (m, 2H), 7.47–7.44 (m, 3H), 7.21–7.17 (m, 3H), 7.13 (m, 2H), 6.98 (m, 1H), 6.90 (m, 2H), 6.82 (m, 2H), 4.80 (s, 2H), 3.27 (d, *J* = 13.2 Hz, 1H), 3.24 (d, *J* = 13.2 Hz, 1H), 2.40 (sp, *J* = 6.8 Hz, 1H), 1.19 (d, *J* = 6.8 Hz, 3H), 0.72 (d, *J* = 6.8 Hz, 3H). ^13^C-NMR (150 MHz, CDCl_3_) δ 178.7, 164.1, 163.3, 149.3, 137.2, 135.7, 135.1, 130.8, 129.7, 128.5, 128.4, 128.3, 128.0 (signal overlapping), 127.4, 126.7, 119.5, 57.6, 42.5, 40.8, 35.4, 17.8, 17.7. HRMS calcd. for C_29_H_27_N_3_O [M˙]^+^ 433.2154; found 433.2102.

*2-Ethyl-5-methyl-5-(1-methylethyl)-5*,*7-dihydro-6H-pyrrolo[2*,*3-d]pyrimidin-6-one* (**28**). To a mixture of BuLi in hexane (2.51 mL, 6.27 mmol, 3.0 eq., 2.5 M) and THF (3 mL), the solution of 2-methyl-5-(1-methylethyl)-5,7-dihydro-6*H*-pyrrolo[2,3-*d*]pyrimidin-6-one (**24b**, 400 mg, 2.09 mmol) in THF (12 mL) was added dropwise at −20 °C under Ar atmosphere. The temperature was allowed to warm to room temperature and the mixture was stirred for 30 min, then MeI (0.39 mL, 0.89 g, 6.27 mmol, 3.0 eq.) in THF (1 mL) was added dropwise. The stirring was continued for further 2 h. The mixture was quenched with saturated NH_4_Cl solution (10 mL), water (5 mL) was added, then the mixture was stirred for 15 min under Ar atmosphere. The layers were separated and the aqueous layer was extracted with EtOAc (3 × 15 mL). The combined organic layer was washed with brine (30 mL), dried over anhydrous MgSO_4_, filtered and the solvent was removed in vacuo at 40 °C. The remaining yellow crystals were purified by gradient elution column chromatography using DCM and DCM/MeOH = 9/1 as the eluents. The product was triturated in hexane (3 mL), washed with DIPE (2 × 1 mL) and dried to give **28** (50 mg, 11%) as colorless crystals, m.p. 149–151 °C. IR (KBr) ν 1743, 1620, 1460, 1193 cm^−1^. ^1^H-NMR (600 MHz, DMSO-*d*_6_) δ 11.37 (br s, 1H), 8.34 (s, 1H), 2.77 (q, *J* = 7.6 Hz, 2H), 2.01 (sp, *J* = 6.8 Hz, 1H), 1.30 (s, 3H), 1.24 (t, *J* = 7.6 Hz, 3H), 0.98 (d, *J* = 6.8 Hz, 3H), 0.66 (d, *J* = 6.8 Hz, 3H). ^13^C-NMR (150 MHz, DMSO-*d*_6_) δ 181.5, 170.1, 164.0, 149.1, 121.6, 50.6, 34.4, 32.1, 20.7, 17.6, 17.2, 12.6. HSQC: 8.34–149.1; 2.77–32.1; 2.01–34.4; 1.30–20.7; 1.24–12.6; 0.98–17.3; 0.66–17.6. HMBC (*J*_long-range_ = 7 Hz; characteristic cross-peaks): 11.37–121.6; 8.34–170.1, 164.0, 121.6; 2.77–170.1; 1.30–181.5, 121.6; 1.24–170.1. HRMS calcd. for C_12_H_17_N_3_O [M˙]^+^ 219.1372; found 219.1387.

### 3.6. General Procedure V for the Synthesis of Compounds ***29a**–**c***

To a mixture of BuLi in hexane (1.50 mL, 3.75 mmol, 2.5 eq., 2.5 M) and THF (3 mL), the solution of the appropriate 2-substituted 5-isopropyl-1,3-diazaoxindole **24a**–**c** (1.50 mmol) in THF (8 mL) was added dropwise at −78 °C, under argon atmosphere. After the addition was complete, the acetone–dry ice bath was removed, the reaction mixture was allowed to warm to room temperature and the apparatus was opened to the air. The stirring was continued for further 2 days. The mixture was quenched with saturated NH_4_Cl solution (10 mL), water (5 mL) was added, then the layers were separated and the aqueous layer was extracted with EtOAc (3 × 5 mL). The combined organic layer was dried over anhydrous MgSO_4_, filtered and the solvent was removed in vacuo at 40 °C. The residue crystallized upon treatment with hexane/DIPE = 3/1 (7 mL), then it was filtered off, washed with hexane/DIPE = 3/1 (2 × 2 mL) and dried.

*5-Hydroxy-5-(1-methylethyl)-5*,*7-dihydro-6H-pyrrolo[2*,*3-d]pyrimidin-6-one* (**29a**). Method A: This compound was prepared according to General Procedure V. The product **29a** was obtained as off-white crystals (228 mg, 79%), m.p. 175–176 °C. IR (KBr) ν 1760, 1159 cm^−1^. ^1^H-NMR (600 MHz, DMSO-*d*_6_) δ 11.42 (br s, 1H), 8.71 (s, 1H), 8.39 (s, 1H), 6.25 (s, 1H), 2.12 (sp, *J* = 6.9 Hz, 1H), 1.03 (d, *J* = 6.9 Hz, 3H), 0.64 (d, *J* = 6.9 Hz, 3H). ^13^C-NMR (150 MHz, DMSO-*d*_6_) δ 179.3, 164.1, 158.3, 149.8, 122.4, 78.1, 34.4, 16.3, 16.0. HSQC: 8.71–158.3; 8.39–149.8; 2.12–34.4; 1.03–16.0; 16.3. HMBC (*J*_long-range_ = 7 Hz; characteristic cross-peaks): 8.71–164.0, 149.8; 8.39–164.0, 158.3, 122.4; 6.25–122.4, 78.1, 34.4; 1.03–78.1, 34.4, 16.3; 0.64–78.1, 34.4, 16.0. HRMS calcd. for C_9_H_11_N_3_O_2_ [M + H]^+^ 193.0851; found 193.0850. Method B: To a mixture of BuLi in hexane (3.80 mL, 9.44 mmol, 3.3 eq., 2.5 M) and THF (3 mL), the solution of 5-(1-methylethyl)-5,7-dihydro-6*H*-pyrrolo[2,3-*d*]pyrimidin-6-one (**24a**, 500 mg, 2.86 mmol) in THF (8 mL) was added dropwise at −78 °C under Ar atmosphere. The mixture was stirred for 30 min, then the temperature was allowed to warm to –20 °C and MeI (0.59 mL, 1.33 g, 9.44 mmol, 3.3 eq.) in THF (1 mL) was added dropwise. The stirring was continued for further 1.5 h. The mixture was quenched with saturated NH_4_Cl solution (10 mL), and water (5 mL) was added. The layers were separated and the aqueous layer was extracted with EtOAc (3 × 15 mL). The combined organic layer was washed with brine (20 mL), dried over anhydrous MgSO_4_, filtered and the solvent was removed in vacuo at 40 °C. The remaining yellow oil was purified by gradient elution column chromatography using DCM and DCM/MeOH = 9/1 as the eluents. The product was triturated in hexane (2 mL), washed with DIPE (2 × 1 mL) and dried to give **29a** (50 mg, 9%) as colorless crystals. Spectral data were identical with those of the product obtained using Method A.

*5-Hydroxy-2-methyl-5-(1-methylethyl)-5*,*7-dihydro-6H-pyrrolo[2*,*3-d]pyrimidin-6-one* (**29b**). Method A: This compound was prepared according to General Procedure V. The product **29b** was obtained as off-white crystals (194 mg, 62%), m.p. 182–184 °C. IR (KBr) ν 1624, 1432 cm^−1^. ^1^H-NMR (400 MHz, DMSO-*d*_6_) δ 11.28 (br s, 1H), 8.26 (s, 1H), 6.16 (s, 1H), 2.49 (s, 3H), 2.10 (sp, *J* = 6.8 Hz, 1H), 1.03 (d, *J* = 6.8 Hz, 3H), 0.62 (d, *J* = 6.8 Hz, 3H). ^13^C-NMR (100 MHz, DMSO-*d*_6_) δ 179.5, 167.2, 164.3, 149.8, 119.1, 78.0, 34.3, 25.9, 16.4, 16.0. HRMS calcd. for C_10_H_13_N_3_O_2_ [M˙]^+^ 207.1008; found 207.1026. Method B: To a mixture of BuLi in hexane (4.13 mL, 6.60 mmol, 2.2 eq., 1.6 M) and THF (5 mL), the solution of 2-methyl-5-(1-methylethyl)-5,7-dihydro-6*H*-pyrrolo[2,3-*d*]pyrimidin-6-one (**24b**, 574 mg, 3.00 mmol) in THF (14 mL) was added dropwise at −78 °C under Ar atmosphere. The mixture was stirred for 30 min, then MeI (0.41 mL, 0.94 g, 6.60 mmol, 2.2 eq.) in THF (1 mL) was added dropwise. The temperature was allowed to warm to room temperature and stirring was continued for further 5 h. The mixture was quenched with saturated NH_4_Cl solution (10 mL), and water (5 mL) was added. The layers were separated and the aqueous layer was extracted with EtOAc (3 × 15 mL). The combined organic layer was washed with brine (30 mL), dried over anhydrous MgSO_4_, filtered and the solvent was removed in vacuo at 40 °C. The remaining orange oil was purified by gradient elution column chromatography using hexane and EtOAc as the eluents. The product was triturated in hexane (2 mL), washed with DIPE (2 × 1 mL) and dried to give **29b** (100 mg, 16%) as colorless crystals. Spectral data were identical with those of the product obtained using Method A.

*5-Hydroxy-2-phenyl-5-(1-methylethyl)-5*,*7-dihydro-6H-pyrrolo[2*,*3-d]pyrimidin-6-one* (**29c**). Method A: This compound was prepared according to General Procedure V. The product **29c** was obtained as off-white crystals (273 mg, 68%), m.p. 184–185 °C. IR (KBr) ν 1730, 1621, 1167 cm^−1^. ^1^H-NMR (400 MHz, DMSO-*d*_6_) δ 11.49 (br s, 1H), 8.51 (s, 1H), 8.35–8.33 (m, 2H), 7.54–7.51 (m, 3H), 6.24 (s, 1H), 2.16 (sp, *J* = 6.8 Hz, 1H), 1.08 (d, *J* = 6.8 Hz, 3H), 0.68 (d, *J* = 6.8 Hz, 3H). ^13^C-NMR (100 MHz, DMSO-*d*_6_) δ 179.6, 164.8, 163.2, 150.2, 137.3, 131.1, 128.9, 127.9, 120.5, 78.1, 34.4, 16.4, 16.1. HRMS calcd. for C_15_H_15_N_3_O_2_ [M˙]^+^ 269.1164; found 269.1150. Method B: To a mixture of BuLi in hexane (4.13 mL, 6.60 mmol, 2.2 eq., 1.6 M) and THF (15 mL), the solution of 2-phenyl-5-(1-methylethyl)-5,7-dihydro-6*H*-pyrrolo[2,3-*d*]pyrimidin-6-one (**24c**, 760 mg, 3.00 mmol) in THF (12 mL) was added dropwise at −78 °C under Ar atmosphere. The mixture was stirred for 30 min, then the temperature was allowed to warm to –20 °C and MeI (0.22 mL, 0.51 g, 3.60 mmol, 1.2 eq.) in THF (1 mL) was added dropwise. The temperature was allowed to warm to 0 °C and stirring was continued for further 4 h. The mixture was quenched with saturated NH_4_Cl solution (10 mL), and water (5 mL) was added. The layers were separated and the aqueous layer was extracted with EtOAc (3 × 15 mL). The combined organic layer was washed with brine (30 mL), dried over anhydrous MgSO_4_, filtered and the solvent was removed in vacuo at 40 °C. The remaining yellow crystals were purified by gradient elution column chromatography using hexane and EtOAc as the eluents. The product was triturated in hexane (2 mL), washed with DIPE (2 × 1 mL) and dried to give **29c** (64 mg, 8%) as colorless crystals. Spectral data were identical with those of the product obtained using Method A.

*3*,*5-Dibenzyl-5-(1-methylethyl)-3*,*5-dihydro-6H-pyrrolo[2*,*3-d]pyrimidin-6-one* (**30a**). To a mixture of BuLi in hexane (3.20 mL, 5.07 mmol, 3.0 eq., 1.6 M) and THF (3 mL), the solution of 5-(1-methylethyl)-5,7-dihydro-6*H*-pyrrolo[2,3-*d*]pyrimidin-6-one (**24a**, 300 mg, 1.69 mmol) in THF (6 mL) was added dropwise at −20 °C under Ar atmosphere. The temperature was allowed to warm to room temperature and the mixture was stirred for 30 min, then BnBr (0.60 mL, 0.87 g, 5.07 mmol, 3.0 eq.) in THF (1 mL) was added dropwise. The stirring was continued for further 1.5 h. The mixture was quenched with saturated NH_4_Cl solution (10 mL), water (5 mL) was added, then the mixture was stirred for 15 min under Ar atmosphere. The layers were separated and the aqueous layer was extracted with EtOAc (3 × 15 mL). The combined organic layer was washed with brine (30 mL), dried over anhydrous MgSO_4_, filtered and the solvent was removed in vacuo at 40 °C. The remaining yellow oil was purified by gradient elution column chromatography using DCM and DCM/MeOH = 9/1 as the eluents. The product was triturated in hexane (3 mL), washed with DIPE (2 × 1 mL) and dried to give **30a** (95 mg, 16%) as colorless crystals, m.p. 271–272 °C. IR (KBr) ν 1705, 1656, 1464 cm^−1^. ^1^H-NMR (600 MHz, DMSO-*d*_6_) δ 8.61 (d, *J* = 2.0 Hz, 1H), 7.97 (d, *J* = 2.0 Hz, 1H), 7.50 (m, 2H), 7.44 (m, 1H), 7.39 (m, 2H), 6.99 (m, 1H), 6.90 (m, 2H), 6.74 (m, 2H), 5.25 (d, *J* = 14.8 Hz, 1H), 5.19 (d, *J* = 14.8 Hz, 1H), 2.99 (d, *J* = 12.7 Hz, 1H), 2.91 (d, *J* = 12.7 Hz, 1H), 2.02 (sp, *J* = 6.8 Hz, 1H), 1.00 (d, *J* = 6.8 Hz, 3H), 0.67 (d, *J* = 6.8 Hz, 3H). ^13^C-NMR (150 MHz, DMSO-*d*_6_) δ 194.5, 182.0, 153.6, 136.8, 136.1, 132.9, 130.0, 129.2, 128.8, 128.0, 127.5, 127.0, 126.2, 58.8, 57.0, 39.4, 34.3, 17.7_4_, 17.7_3_. COSY (characteristic cross-peaks): 7.50–7.44, 7.39; 2.02–1.00, 0.67. HSQC: 8.61–153.6; 7.97–132.9; 7.50–129.2; 7.44–128.8; 7.39–128.0; 6.99–127.0; 6.90–127.5; 6.74–130.0; 5.25–57.0; 5.19–57.0; 2.99–39.5; 2.91–39.5; 2.02–34.3; 1.00, 0.67–17.7_4_, 17.7_3_. HMBC (*J*_long-range_ = 7 Hz; characteristic cross-peaks): 8.61–182.0, 132.9, 57.0; 7.97–182.0, 153.6, 127.0; 7.39–57.0; 6.74–39.5; 5.25, 5.19–153.6, 136.1, 132.9, 128.0; 2.99–136.8, 130.0; 2.91–136.8, 130.0, 127.0; 2.02–194.5. HRMS calcd. for C_23_H_23_N_3_O [M˙]^+^ 357.1841; found 357.1851.

*3*,*5-Dibenzyl-2-methyl-5-(1-methylethyl)-3*,*5-dihydro-6H-pyrrolo[2*,*3-d]pyrimidin-6-one* (**30b**). To a mixture of BuLi in hexane (1.26 mL, 3.14 mmol, 3.0 eq., 2.5 M) and THF (2 mL), the solution of 2-methyl-5-(1-methylethyl)-5,7-dihydro-6*H*-pyrrolo[2,3-*d*]pyrimidin-6-one (**24b**, 200 mg, 1.05 mmol) in THF (6 mL) was added dropwise at −20 °C under Ar atmosphere. The temperature was allowed to warm to room temperature and the mixture was stirred for 30 min, then BnBr (0.37 mL, 0.54 g, 3.14 mmol, 3.0 eq.) in THF (1 mL) was added dropwise. The stirring was continued for further 2 h. The mixture was quenched with saturated NH_4_Cl solution (10 mL), water (5 mL) was added, then it was stirred for 15 min under Ar atmosphere. The layers were separated and the aqueous layer was extracted with EtOAc (3 × 15 mL). The combined organic layer was washed with brine (30 mL), dried over anhydrous MgSO_4_, filtered and the solvent was removed in vacuo at 40 °C. The remaining orange oil was purified by gradient elution column chromatography using DCM and DCM/MeOH = 9/1 as the eluents. The product was triturated in hexane (3 mL), washed with DIPE (2 × 1 mL) and dried to give **30b** (215 mg, 56%) as off-white crystals, m.p. 250–252 °C. IR (KBr) ν 1757, 1653 cm^−1^. ^1^H-NMR (600 MHz, DMSO-*d*_6_) δ 9.11 (br s, 1H), 7.53 (m, 2H), 7.46 (m, 1H), 7.29 (m, 2H), 7.15 (m, 1H), 7.12 (m, 2H), 6.98 (m, 2H), 5.71 (d, *J* = 16.0 Hz, 1H), 5.64 (d, *J* = 16.0 Hz, 1H), 3.32 (d, *J* = 13.1 Hz, 1H), 3.18 (d, *J* = 13.1 Hz, 1H), 2.61 (s, 3H), 2.27 (sp, *J* = 6.8 Hz, 1H), 1.10 (d, *J* = 6.8 Hz, 3H), 0.85 (d, *J* = 6.8 Hz, 3H). ^13^C-NMR (150 MHz, DMSO-*d*_6_) δ 183.4 (br), 170.9 (br), 165.2, 142.8 (br), 135.7, 134.1, 129.9, 129.4, 128.9, 128.2, 127.4, 127.1, 122.8, 58.7, 58.5, 39.4, 34.9, 23.1, 17.6, 17.5. HSQC: 9.11–142.8; 7.53–129.4; 7.46–128.9; 7.29–127.4; 7.15–127.2; 7.12–128.2; 6.98–129.9; 5.71–58.5; 5.64–58.5; 3.32–39.4; 3.18–39.4; 2.61–23.1; 2.27–34.9; 1.10–17.6; 0.85–17.5. HMBC (*J*_long-range_ = 7 Hz; characteristic cross-peaks): 9.11–170.9, 165.2, 122.8, 58.5; 7.53–134.1; 7.29–58.5; 7.12–135.7; 5.71, 5.64–165.2, 142.8, 134.1; 3.32–183.4; 3.18–183.4, 122.8; 2.61–165.2. HRMS calcd. for C_24_H_25_N_3_O [M˙]^+^ 371.1998; found 371.1994.

*3*,*5*,*7-Tribenzyl-5-(1-methylethyl)-6-oxo-6*,*7-dihydro-5H-pyrrolo[2*,*3-d]pyrimidin-3-ium bromide* (**31a**). Method A: A mixture of **27d** (340 mg, 0.95 mmol), BnBr (0.43 mL, 0.62 g, 3.80 mmol) and acetonitrile (3 mL) was stirred for 27 h at room temperature. The solvent was removed at 40 °C in vacuo and the crystals was triturated with hexane (5 mL), filtered off, washed with DIPE (3 × 2 mL) and dried. The product **31a** was obtained as colorless crystals (431 mg, 86%), m.p. 220–222 °C (*i*-PrOH). IR (KBr) ν 1728, 1625, 1410 cm^−1^. ^1^H-NMR (600 MHz, DMSO-*d*_6_) δ 9.65 (d, *J* = 1.6 Hz, 1H), 9.36 (d, *J* = 1.6 Hz, 1H), 7.59 (m, 2H), 7.56 (m, 2H), 7.51 (m, 1H), 7.26 (m, 3H), 7.02 (m, 1H), 6.94 (m, 2H), 6.80 (m, 2H), 6.63 (m, 2H), 5.73 (d, *J* = 14.4 Hz, 1H), 5.67 (d, *J* = 14.4 Hz, 1H), 4.85 (d, *J* = 15.3 Hz, 1H), 4.80 (d, *J* = 15.3 Hz, 1H), 3.42 (d, *J* = 13.2 Hz, 1H), 3.25 (d, *J* = 13.2 Hz, 1H), 2.41 (sp, *J* = 6.9 Hz, 1H), 1.04 (d, *J* = 6.9 Hz, 3H), 0.86 (d, *J* = 6.9 Hz, 3H). ^13^C-NMR (150 MHz, DMSO-*d*_6_) δ 178.0, 166.1, 156.3, 143.0, 134.5, 134.4, 134.2, 129.7, 129.6, 129.4, 128.9, 128.8, 128.3, 128.1, 127.7, 127.2, 123.8, 59.9, 58.2, 43.1, 39.2, 35.4, 17.7, 17.2. COSY (characteristic cross-peaks): 7.26–6.94; 7.02–6.80; 6.80–6.63; 3.42–3.25; 2.41–1.04, 0.86. HSQC: 9.65–156.3; 9.36–143.0; 7.59–128.9; 7.56–129.5; 7.51–129.6; 7.26–128.8, 128.1; 7.02–127.2; 6.94–127.7; 6.63–129.7; 5.73–59.9; 5.67–59.9; 4.85–43.1; 4.80–43.1; 3.42–39.2; 3.25–39.2; 2.41–35.4; 1.04–17.7; 0.86–17.2. HMBC (*J*_long-range_ = 7 Hz; characteristic cross-peaks): 9.65–166.1, 143.0, 59.9; 9.36–166.1, 156.3, 123.8, 59.9; 7.59–59.9; 7.56–134.4; 7.26–134.2; 6.94–43.1; 6.80–134.5; 6.63–39.2; 2.41–123.8, 178.0; 1.04–58.2; 0.86–58.2. COSY: 7.26–6.94; 7.02–6.80; 6.80–6.63; 3.42–3.25; 2.41–1.04, 0.86. HSQC: 9.65–156.3; 9.36–143.0; 7.59–128.9; 7.56–129.5; 7.51–129.6; 7.26–128.8, 128.1; 7.02–127.2; 6.94–127.7; 6.63–129.7; 5.73–59.9; 5.67–59.9; 4.85–43.1; 4.80–43.1; 3.42–39.2; 3.25–39.2; 2.41–35.4; 1.04–17.7; 0.86–17.2. Selective NOESY: 9.36–7.59, 6.63, 5.73, 5.67, 3.42, 1.04; 9.65–7.59, 5.73, 5.67. HRMS calcd. for C_30_H_30_N_3_O [M]^+^ 448.238; found 448.238. Elementary analysis of C_30_H_30_BrN_3_O calcd. for Br: 15.12%; found: 14.80%. Method B: To a mixture of BuLi in hexane (3.20 mL, 5.07 mmol, 3.0 eq., 1.6 M) and THF (3 mL), the solution of 5-(1-methylethyl)-5,7-dihydro-6*H*-pyrrolo[2,3-*d*]pyrimidin-6-one (**24a**, 300 mg, 1.69 mmol) in THF (6 mL) was added dropwise at −20 °C, under Ar atmosphere. The temperature was allowed to warm to room temperature and the mixture was stirred for 30 min, then BnBr (0.60 mL, 0.87 g, 5.07 mmol, 3.0 eq.) in THF (1 mL) was added dropwise. The stirring was continued for further 1.5 h. The mixture was quenched with saturated NH_4_Cl solution (10 mL), water (5 mL) was added, then it was stirred for 15 min under Ar atmosphere. The layers were separated and the aqueous layer was extracted with EtOAc (3 × 15 mL). The combined organic layer was washed with brine (30 mL), dried over anhydrous MgSO_4_, filtered and the solvent was removed in vacuo at 40 °C. The remaining yellow oil was purified by gradient elution column chromatography using DCM and DCM/MeOH = 9/1 as the eluents. The product was triturated in hexane (3 mL), washed with DIPE (2 × 1 mL) and dried to give **30a** (110 mg, 12%) as colorless crystals. Spectral data were identical with those of the product obtained using Method A.

*3*,*5*,*7-Tribenzyl-2-methyl-5-(1-methylethyl)-6-oxo-6*,*7-dihydro-5H-pyrrolo[2*,*3-d]pyrimidin-3-ium bromide* (**31b**). Method A: A mixture of **27e** (371 mg, 1.00 mmol), BnBr (0.45 mL, 0.65 g, 3.80 mmol) and acetonitrile (3 mL) was stirred for 116 h at room temperature. The solvent was removed at 40 °C in vacuo and the crystals was triturated with hexane (5 mL), filtered off, washed with DIPE (3 × 2 mL) and dried. The product **31b** was obtained as off-white crystals (386 mg, 71%), m.p. 104–106 °C (*i*-PrOH). IR (KBr) ν 1756, 1651 cm^−1^. ^1^H-NMR (600 MHz, DMSO-*d*_6_) δ 9.36 (s, 1H), 7.55 (t, *J* = 7.4 Hz, 2H), 7.48 (t, *J* = 7.4 Hz, 1H), 7.36 (d, *J* = 7.4 Hz, 2H), 7.27–7.26 (m, 3H), 7.07 (t, *J* = 7.4 Hz, 1H), 7.02–6.99 (m, 4H), 6.86 (d, *J* = 7.2 Hz, 2H), 5.79 (d, *J* = 15.8 Hz, 1H), 5.71 (d, *J* = 15.8 Hz, 1H), 4.84 (d, *J* = 15.2 Hz, 1H), 4.79 (d, *J* = 15.2 Hz, 1H), 3.44 (d, *J* = 13.3 Hz, 1H), 3.29 (d, *J* = 13.3 Hz, 1H), 2.77 (s, 3H), 2.39 (sp, *J* = 6.8 Hz, 1H), 1.08 (d, *J* = 6.8 Hz, 3H), 0.82 (d, *J* = 6.8 Hz, 3H). ^13^C-NMR (150 MHz, CDCl_3_) δ 178.3, 166.6, 165.8, 145.0, 134.8, 134.3, 133.4, 129.7, 129.4, 129.2, 128.8, 128.5, 128.1, 127.9, 127.8, 127.4, 121.5, 59.3, 58.0, 43.0, 39.2, 35.6, 23.5, 17.6, 17.3. HRMS calcd. for C_31_H_32_N_3_O [M]^+^ 462.254; found 462.253. Elementary analysis of C_31_H_32_BrN_3_O calcd. for Br: 14.73%; found: 14.80%. Method B: To a mixture of BuLi in hexane (1.26 mL, 3.14 mmol, 3.0 eq., 2.5 M) and THF (2 mL), the solution of 2-methyl-5-(1-methylethyl)-5,7-dihydro-6*H*-pyrrolo[2,3-*d*]pyrimidin-6-one (**24b**, 200 mg, 1.05 mmol) in THF (6 mL) was added dropwise at −20 °C, under Ar atmosphere. The temperature was allowed to warm to room temperature and the mixture was stirred for 30 min, then BnBr (0.37 mL, 0.54 g, 3.14 mmol, 3.0 eq.) in THF (1 mL) was added dropwise. The stirring was continued for further 2 h. The mixture was quenched with saturated NH_4_Cl solution (10 mL), water (5 mL) was added, then it was stirred for 15 min under Ar atmosphere. The layers were separated and the aqueous layer was extracted with EtOAc (3 × 15 mL). The combined organic layer was washed with brine (30 mL), dried over anhydrous MgSO_4_, filtered and the solvent was removed in vacuo at 40 °C. The remaining orange oil was purified by gradient elution column chromatography using DCM and DCM/MeOH = 9/1 as the eluents. The product was triturated in hexane (3 mL), washed with DIPE (2 × 1 mL) and dried to give **31b** (108 mg, 19%) as off-white crystals. Spectral data were identical with those of the product obtained using Method A.

## 4. Conclusions

The alkylation reactions of *N*(7)-unsubstituted 1,3-diazaoxindoles have been investigated using the 5-isopropyl derivatives as the model compounds. Alkylation of 5-isopropyl derivatives **24** (bearing various substituents at the *C*(2) position of the pyrimidine ring) with MeI or BnBr in the presence of NaOH at room temperature led to the *C*(5),*N*(7)-disubstituted analogues **27**. When BuLi was used as the base and the alkylating agent was added at −20 °C, a selective *C*(5)-alkylation could be carried out resulting in derivatives **26**. Some interesting by-products were also identified in the above reactions. When methylation of the lithium salt of 2-methyl derivative **24b** was performed at ambient temperature, methylation of the 2-methyl moiety was also observed giving rise to the formation of 2-ethyl derivative **28**. The insufficient pre-inertization of the lithiation reactions led to 5-hydroxy by-products **29**. During the benzylation of 2-unsubstituted (**24a**) and 2-methyl-1,3-diazaoxindole (**24b**) with BuLi and BnBr at room temperature, in addition to *C*(5)-benzylated derivatives, the unexpected *N*(3),*C*(5)-dibenzylated compounds **30** and *N*(3),*C*(5),*N*(7)-tribenzylated quaternary bromide salts **31** were also isolated. The structure of the latter was proven by single-crystal X-ray diffraction and a detailed spectroscopic analysis. The present study may help other research groups perform various alkylation reactions of 1,3-diazaoxindoles.

## Figures and Tables

**Table 1:** Yields of the 5,7-dialkylation reactions of 5-isopropyl-1,3-diazaoxindoles **24a**–**c**.

Entry	24	R^1^	R^2^	X	27	Yield of 27 (%)
1	**a**	H	Me	I	**a**	64
2	**b**	Me	Me	I	**b**	80
3	**c**	Ph	Me	I	**c**	91
4	**a**	H	Bn	Br	**d**	70
5	**b**	Me	Bn	Br	**e**	82
6	**c**	Ph	Bn	Br	**f**	82

**Table 2:** Isolated products of the methylation reactions of 5-isopropyl-1,3-diazaoxindoles **24** in the presence of a large excess of BuLi and MeI.

Entry	24	R^1^	Yield of 26 (%)	Yield of 27 (%)
1	**a**	H	75	2
2	**b**	Me	42	22
3	**c**	Ph	56	31

**Table 3:** Isolated products of the benzylation reactions of 5-isopropyl-1,3-diazaoxindoles **24a**,**b** in the presence of a large excess of BuLi and BnBr.

Entry	24	R^1^	26	Yield of 26 (%)	30	Yield of 30 (%)	31	Yield of 31 (%)
1	**a**	H	**d**	36	**a**	16	**a**	12
2	**b**	Me	**e**	5	**b**	56	**b**	19

## Supplementary Materials

CCDC 1532352 contains the supplementary crystallographic data for this paper (X-Ray structure, conditions of the single-crystal X-ray measurements and CIF structure file for compound **31a**). These data can be obtained free of charge via http://www.ccdc.cam.ac.uk/conts/retrieving.html (or from the CCDC, 12 Union Road, Cambridge CB2 1EZ, UK; Fax: +44 1223 336033; E-mail: deposit@ccdc.cam.ac.uk).
