# Supplementary figures and images for: Study on the Alkylation Reactions of N(7)-Unsubstituted 1,3-Diazaoxindoles
Source: Molecules. 2017 May 19;22(5):846. doi: 10.3390/molecules22050846 (PMC6154441; doi:10.3390/molecules22050846)

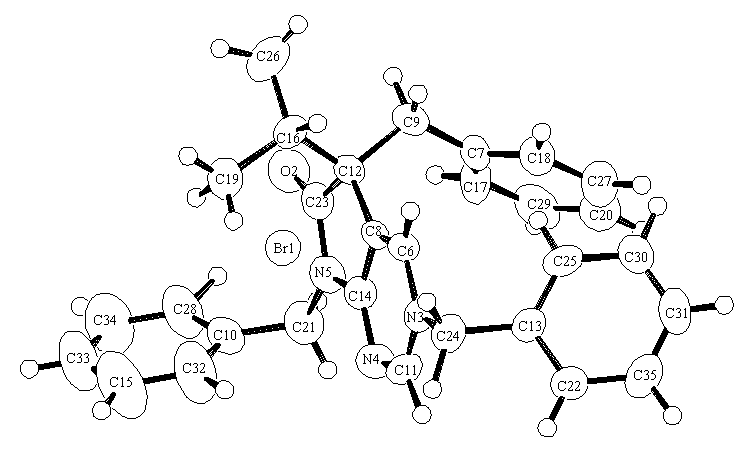

Supplement: Supplementary file 1 [file molecules-22-00846-s001.zip › compound 31a ORTEP.bmp]
